# Antibiotic target discovery by integrated phenotypic and activity-based profiling of electrophilic fragments

**DOI:** 10.1016/j.chembiol.2025.02.001

**Published:** 2025-02-27

**Authors:** Yizhen Jin, Sadhan Jana, Mikail E. Abbasov, Hening Lin

**Affiliations:** 1 Graduate Program of Biochemistry, Molecular and Cell Biology, Department of Molecular Biology and Genetics, Cornell University, Ithaca, NY 14853, USA; 2 Department of Chemistry and Chemical Biology, Cornell University, Ithaca, NY 14853, USA; 3 Department of Medicine and Department of Chemistry, The University of Chicago, 900 E. 57^th^ Street, Chicago, IL 60637, USA; 4 Howard Hughes Medical Institute, Department of Chemistry and Chemical Biology, Department of Molecular Biology and Genetics, Cornell University, Ithaca, NY 14853, USA; 5 Howard Hughes Medical Institute, Department of Medicine and Department of Chemistry, The University of Chicago, 900 E. 57^th^ Street, Chicago, IL 60637, USA; 6 Lead contact

## Abstract

The emergence of antibiotic resistance necessitates the discovery of novel bacterial targets and antimicrobial agents. Here, we present a bacterial target discovery framework that integrates phenotypic screening of cysteine-reactive fragments with competitive activity-based protein profiling to map and functionally characterize the targets of screening hits. Using this approach, we identify β-ketoacyl-acyl carrier protein synthase III (FabH) and MiaA tRNA prenyltransferase as primary targets of a hit fragment, 10-F05, that confer bacterial stress resistance and virulence in *Shigella flexneri*. Mechanistic investigations elucidate that covalent C112 modification in FabH, an enzyme involved in bacterial fatty acid synthesis, results in its inactivation and consequent growth inhibition. We further demonstrate that irreversible C273 modification at the MiaA RNA-protein interaction interface abrogates substrate tRNA binding, attenuating resistance and virulence through decreased translational accuracy. Our findings underscore the efficacy of integrating phenotypic and activity-based profiling of electrophilic fragments to accelerate the identification and pharmacologic validation of new therapeutic targets.

## In brief

Jin et al. present an antibiotic target discovery framework that integrates phenotypic screening of cysteine-reactive fragments with competitive activity-based protein profiling to functionally characterize the targets of identified hits. The hit fragment, 10-F05, covalently modifies Cys112 of FabH and Cys273 of MiaA to inhibit bacterial growth, stress resistance, and virulence.

## Graphical abstract

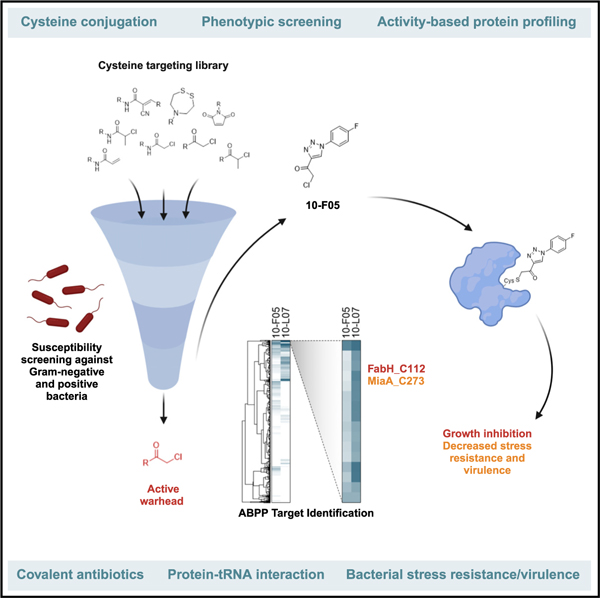

## INTRODUCTION

Antibiotics are crucial for human medicine, yet the rise of antibiotic resistance jeopardizes their efficacy. To address this issue, one approach involves targeting the mechanisms behind resistance, such as proteins responsible for degrading or exporting antibiotics. Another method seeks to broaden the target scope by developing antibiotics that act on novel bacterial targets. Most existing antibiotics target a narrow range of well-understood mechanisms, including the synthesis of cell walls, proteins, and nucleic acids.^1^ Consequently, antibiotics that target new bacterial processes could offer effective treatments for infections caused by bacteria that have developed resistance to current treatments.

To rapidly identify novel antibiotic targets and develop leading compounds for them, an expedient and effective strategy is essential. Inspired by the recent successful development of KRAS G12C inhibitor to target proteins that are traditionally considered as “undruggable”^2^ and the existence of antibiotics that covalently target proteins (e.g., penicillin), we hypothesized that screening a cysteine-reactive compound library can expand the pool of druggable proteins in bacterial proteomes. This method leverages covalent interactions to engage proteins that are otherwise overlooked in traditional drug screening efforts. Moreover, the use of competitive activity-based protein profiling (ABPP) harnesses the covalent bond formation with hit compounds to simplify the complex task of elucidating targets.^3–5^ By integrating these methodologies, we aim to expedite the identification and characterization of novel targets for antibiotic discovery and the identification of lead compounds for these targets.

Despite the growing interest in compounds targeting cysteine residues,^6–8^ most previous studies have relied on small libraries, often limited to a few types of cysteine-reactive agents like chloroacetamide.^9,10^ This reliance on small libraries may impede the discovery of proteins that could be potential drug targets. Here, we applied a much larger and diverse library aimed at cysteine targeting, which comprises 3,200 fragment-like covalent ligands, for antibacterial screening. This approach helped us uncover new potential targets and lead compounds. Among these, one lead compound, 10-F05, demonstrated broad-spectrum antibacterial effectiveness. Through competitive activity-based protein profiling (ABPP), we identified and validated two proteins, β-ketoacyl-acyl carrier protein (ACP) synthase III (FabH) and MiaA, as physiologically relevant targets of 10-F05. Notably, MiaA has not been previously recognized as a target for antibiotics development, underscoring the advantage of integrating phenotypic screening of covalent compound libraries with ABPP to discover novel targets and their corresponding lead compounds.

## RESULTS

### Screening of a cysteine-reactive compound library identifies antibacterial hits

To expand the druggable targets within bacterial cysteinome, we obtained a diverse cysteine-targeting compound library (Cys-library) consisting of 3,200 fragment-like compounds from Enamine (Figures S1A–S1F). Initially, we assessed the cytotoxicity of this library in HEK293T cells at a concentration of 25 µM over 2 days (Figure S1G). In general, chloromethyl ketone and chloroacetamide scaffolds exhibited higher cytotoxicity in HEK293T cells compared to other warhead scaffolds, while 2-chloropropionamide and 2-chloroethyl ketone scaffolds displayed higher tolerance in the mammalian cells. However, the level of cytotoxicity varied among compounds; notably, some chloromethyl ketone-based compounds exhibited low cytotoxicity in HEK293T cells. These data will later assist in refining our selection of antibacterial compounds for more detailed characterization.

**Figure 1.**
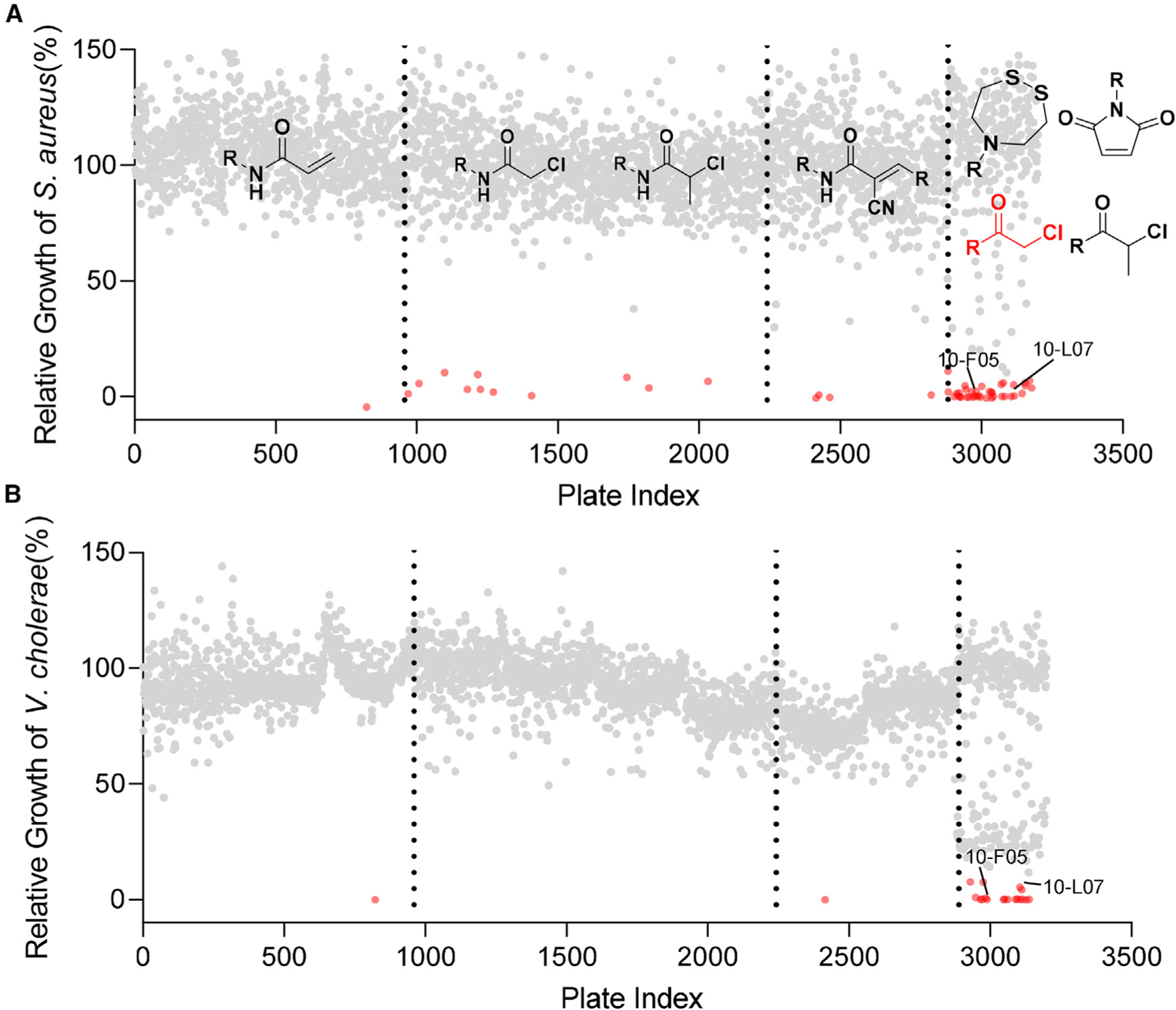
Bacterial growth inhibition screen using an electrophilic cysteine-reactive fragment library at 25 µM Relative bacterial growth was determined by normalizing the OD600 value to the DMSO control. (A) Growth inhibition screen in *S. aureus* (MSSA476). (B) Growth inhibition screen in *V. cholerae* (SAD30). Hits (relative growth ≤10%) are labeled in red.

Next, we screened the Cys-library to identify active compounds against *S. aureus* (MSSA476) and *V. cholerae* (SAD30) at 25 µM (Figures 1A and 1B). The initial screening resulted in the identification of 48 and 17 compounds that inhibited the growth of *S. aureus* and *V. cholerae* in liquid culture, respectively (Table S1). Notably, several compounds that were effective against *S. aureus* did not show activity against *V. cholerae*. This discrepancy may be attributed to the differences in cell wall composition or cysteine targets between the two strains.^10,11^ From these hits, we excluded 11 that showed high cytotoxicity in HEK293T cells at 25 µM.^10,12^ Additionally, two compounds (3-J04 and 8-I07) were excluded from further analysis due to the presence of a nitrofuran moiety, which is known for its toxicity.^13^ The remaining chloromethyl ketone hits were evaluated to measure their minimum inhibitory concentrations (MICs) against an expanded collection of pathogens including *S. flexneri* M90T, *V. cholerae* SAD30, *E. coli* JPN15, and *S. aureus* MSSA476 (Table 1). Most chloromethyl ketone hits displayed MICs ranging from 5 to 50 µM, with slight variations observed among the four pathogens.

We employed a reduced 5,5ʹ-dithiobis-(2-nitrobenzoic acid) (Ellman’s reagent) assay to profile the reactivity of Cys-library (Figure S2A).^14^ Most compounds could be fitted with a second-order reaction model (Figure S2B). Chloromethyl ketone exhibited the highest reactivity among all the warheads tested (Figure S2C). Interestingly, the thiol reactivity did not correlate with the antibacterial activity. For instance, 10-C09 and 10-F05 exhibited similar reactivity toward thiols, yet they displayed a 2.5- to 10-fold difference in their MICs across the four tested pathogens. Furthermore, two derivatives of 10-F05, 10-M19 and 10-L07, which are 1.2-fold and 2.5-fold more reactive, respectively, exhibited 2- to 10-fold worse MICs compared to 10-F05 (Figure S2D). Overall, these findings emphasized the significance of non-covalent moieties of 10-F05 in facilitating target engagement.

### 10-F05 is effective against multiple ESKAPE pathogens and demonstrates a slow resistance development

Among the chloromethyl ketone hits identified, 10-F05 emerged as the most effective against the majority of the bacterial strains tested (Table 1). Consequently, we pursued additional experiments with 10-F05. We found that 10-F05 inhibits multiple ESKAPE pathogens, including methicillin-resistant *S. aureus* (MRSA), *E. cloacae*, *K. pneumoniae*, and *E. coli* (Table S5). Nonetheless, its efficacy was significantly reduced against several multidrug-resistant gram-negative strains (*K. pneumoniae*, *A. baumannii*, and *P. aeruginosa*. The antibiotic spectrum for bacterial strains used is reported in Table S9).

**Figure 2.**
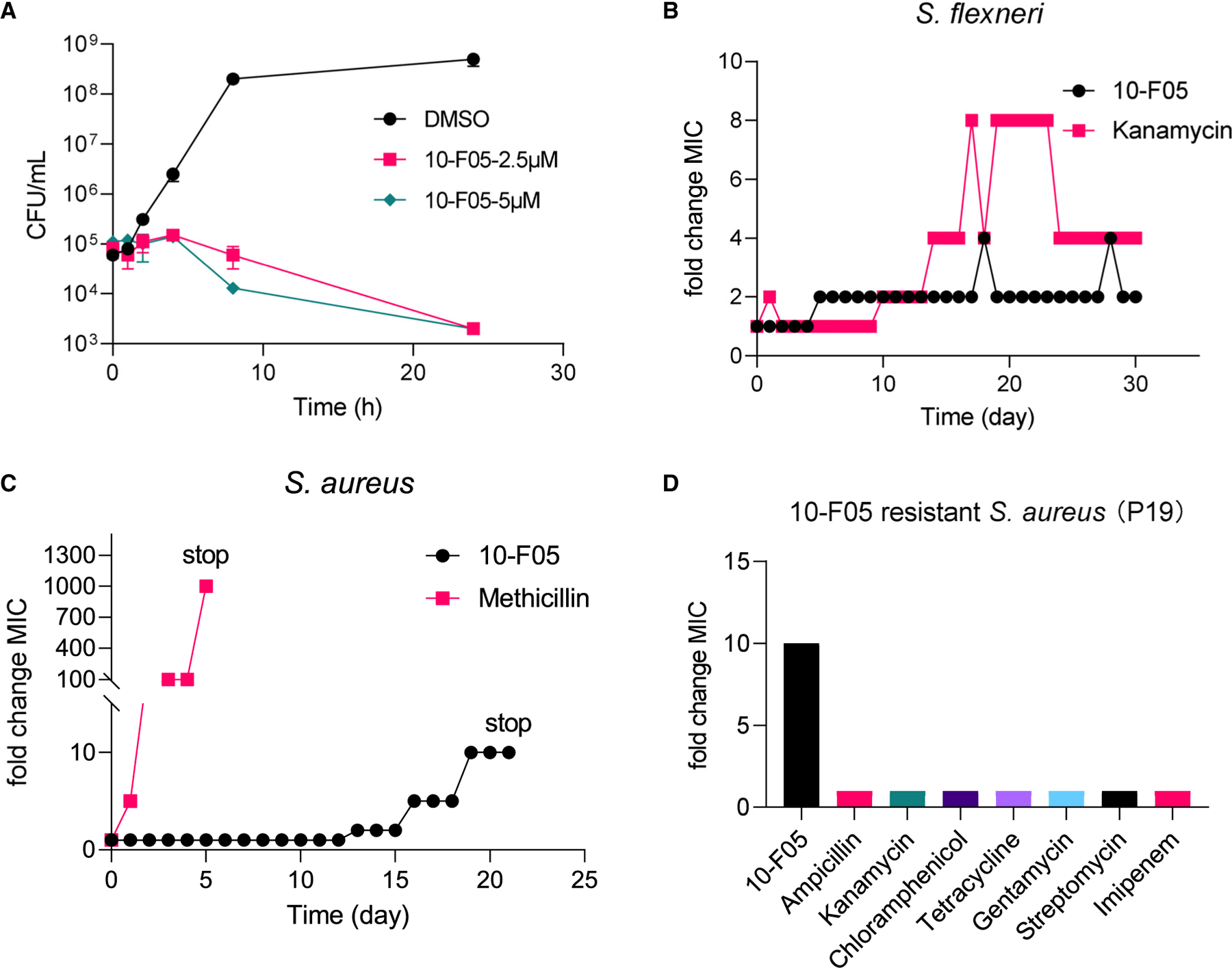
Antibacterial activity of and resistance development to 10-F05 (A) Time-dependent killing of *S. flexneri* M90T by 10-F05 (*n* = 2, mean ± SD). (B) Resistance development of *S. flexneri* M90T to 10-F05 and kanamycin during daily serial passaging with sub-MIC concentrations. (C) Resistance development of *S. aureus* MSSA476 to 10-F05 and methicillin during daily serial passaging with sub-MIC concentrations. (D) MIC fold change of the 10-F05-resistant *S. aureus* MSSA476 toward other classes of antibiotics. MIC values were reported in supplementary data.

Further investigations into 10-F05’s bacterial killing properties were conducted through a time-dependent killing experiment, starting with an inoculum of approximately 10^5^ colony-forming unit/mL in LB medium. These results showed that 2.5 µM of 10-F05 eliminates ~99% of *S. flexneri* within a 24-h period (Figure 2A). Additionally, 10-F05 was found to exhibit low cytotoxicity in HEK293T cells when compared to other chloromethyl ketone hits (Figure S1H). The 50% inhibition concentration (IC_50_ value) of 10-F05 was ~50 µM in both HEK293T and A549 cells after a 2-day incubation period (Figure S1I). The selectivity ratio ranged from 2 to 20, depending on the bacterial strains.

Our data showed that 10-F05 acted as bactericidal in *S. flexneri* while bacteriostatic in *S. aureus* MSSA476 (Figure S3). Next, we investigated the development of resistance against 10-F05 in *S. aureus* MSSA476 and *S. flexneri* M90T (Figures 2B and 2C). Given the presumption that fragment-like hits potentially engage multiple bacterial proteins, we anticipated a more challenging path for bacterial resistance development. To test this, *S. flexneri* and *S. aureus* were passaged daily with sub-MIC concentrations of 10-F05 or two known antibiotics (kanamycin and methicillin). The bacteria that survived each day were then analyzed to determine the MIC values of these compounds. *S. flexneri* M90T displayed a 2-fold increase in MIC to 10-F05 on day 5 (MIC = 5 µM) and maintained a less than 4-fold change in MIC after 30 days of sub-MIC exposure. During this period, *S. flexneri* M90T developed a higher resistance to kanamycin, with a maximum 8-fold increase in MIC (Figure 2B). Conversely, *S. aureus* quickly developed resistance to methicillin, exhibiting a greater than 200-fold increase in MIC after just 5 days (MIC > 1 mg/mL) (Figure 2C). In contrast, resistance development to 10-F05 in *S*. *aureus* occurred at a much slower rate, starting on day 13 and reaching a 10-fold change in MIC by day 19 (Figure 2C). This observation aligns with the frequency of resistance profiles of *S. flexneri* M90T and *S. aureus* MSSA476 against 10-F05 (Figure S3). The gradual resistance development rate for 10-F05 in both *S. flexneri* and *S. aureus* supports our hypothesis that a poly-pharmacological mechanism of 10-F05 impedes resistance development. Notably, the 10-F05-resistant *S. aureus* strains (Figure 2D) and the *S. flexneri* P30 strain (Table S5) did not exhibit significant cross-resistance to other commonly used antibiotic classes, suggesting a novel mechanism of action of 10-F05.

### Competitive ABPP identifies FabH, MiaA, and PdxY as targets of 10-F05

Encouraged by its promising activity in both gram-positive and gram-negative bacteria, we aimed to identify the protein targets of 10-F05. To verify that 10-F05 and similar chloromethyl ketone compounds inhibit bacterial growth by targeting the bacterial cysteinome, we evaluated the MICs of their respective negative control compounds, where the cysteine-reactive chloromethyl ketone group was substituted with a methyl ketone group, against model pathogens (Table S2). The control compounds showed no activity, indicating that the growth inhibitory effect is due to the covalent modification of bacterial proteins. This suggests that cysteine-centric, competitive ABPP would be an effective method for target identification.

**Figure 3.**
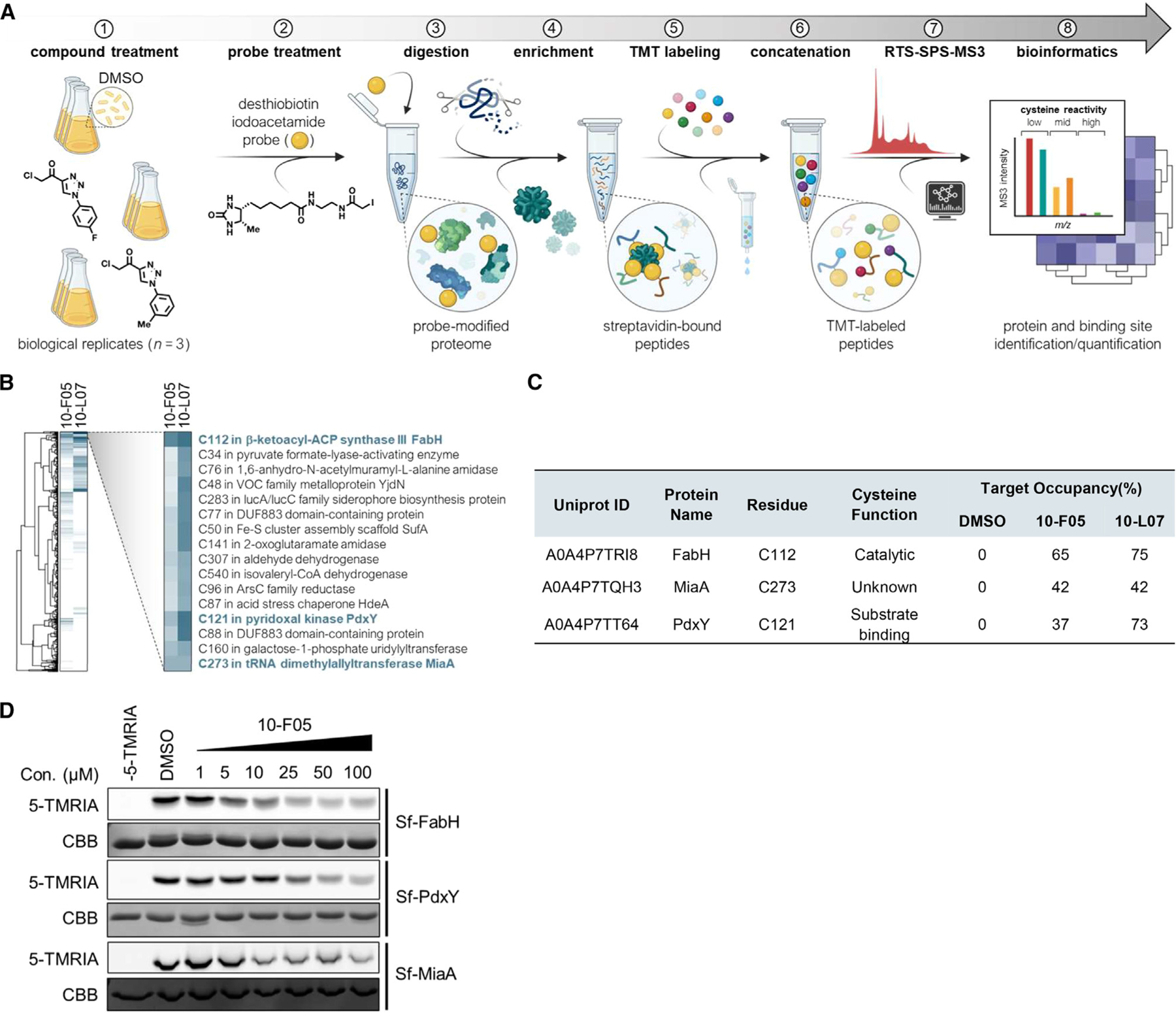
Competitive activity-based protein profiling identified FabH, MiaA, and PdxY as targets of 10-F05 (A) Schematic of TMTpro-18plex-based workflow for mapping cysteine-drug interactions in live *S. flexneri*. (B) Heatmap analysis of the protein targets of 10-F05 and 10-L07. (C) Target occupancy table of highlighted cysteines in (B). Cysteine function is annotated based on UniProt database. (D) In-gel fluorescence labeling of three purified *S. flexneri* target proteins. Purified proteins were incubated with 10-F05 at indicated concentrations for 1 h and labeled by 5-TMRIA to visualize the unbound cysteines. Uncropped gel images are shown in supporting information.

We then applied ABPP in *S. flexneri* M90T to identify potential targets of 10-F05. In addition to 10-F05, we included its analog, 10-L07, which possesses similar antibacterial activity, in our target identification process to enhance confidence in the identified targets. Live *S. flexneri* M90T cells suspended in PBS were treated with 10-F05, 10-L07, or DMSO. After lysis, the cells were treated with iodoacetamide-conjugated desthiobiotin (IA-DTB). Following digestion with trypsin and Lys-C, IA-DTB-labeled peptides were affinity enriched and barcoded with tandem mass tags. Real-time search coupled with synchronous precursor selection and MS3 fragmentation was implemented to optimize both quantification and proteomic coverage (Figure 3A).^15^

We identified 1,035 cysteines across 588 proteins out of 3,986 total proteins in *S. flexneri*, which is comparable to a report from the Hacker group (Figure S4A).^10^ Gene ontology for the identified proteins showed no apparent enrichment (Figure S4B). We next annotated the identified proteins using NetGenes database to predict the essentiality of the identified proteins.^16^ Among the 588 identified proteins, 103 were predicted to be likely essential in *S. flexneri* M90T (Figure S4C). Subsequently, we focused on the cysteines engaged by 10-F05 and 10-L07. FabH Cys112 was identified as the top hit for both 10-F05 and 10-L07, exhibiting target occupancy of 65% and 75%, respectively (Figures 3B and 3C). FabH converts malonyl-ACP to acetoacyl-ACP in bacterial fatty acid biosynthesis. Its essential role has been underscored by multiple studies in *S. aureus*.^4,17–19^ However, the deletion of *fabH* is not lethal in *E. coli* strains.^20^ Several potent FabH inhibitors have demonstrated activity against MRSA.^4,19,21,22^ Among those, Oxa2 was found to bind FabH Cys112, the same cysteine identified in our proteomic result. Oxa2 showed an MIC range from 0.25 to 0.5 µg/mL (1–2 µM) against a panel of *S. aureus*. However, Oxa2 lacks activity against gram-negative strains, while 10-F05 is effective against both gram-negative and gram-positive strains. The identification of FabH, a validated antibiotic target, as a target of 10-F05 clearly illustrates the utility of integrating phenotypic screening of covalent compound libraries with competitive ABPP in discovering new targets for antibiotics development.

The next two cysteine targets identified for 10-F05 are MiaA Cys273 and PdxY Cys121. These cysteines exhibit slightly lower target occupancy compared to that of FabH Cys112 (Figure 3C). MiaA generates the i^6^A-37 tRNA by catalyzing the addition of an isopentenyl group onto the N6-nitrogen of Ade-37 next to the anticodon. The product is subsequently methylthiolated by the radical-S-adenosylmethionine enzyme MiaB to yield ms^2^i^6^A-37.^23^ This modification is known to enhance the interaction of tRNA with UNN codons (Phe, Leu, Ser, Tyr, Cys, and Trp), thereby promoting reading frame maintenance and translational fidelity.^24,25^ Moreover, MiaA is essential for bacterial fitness and virulence in diverse host niches.^23^ Intraperitoneally injected *miaA* KO pathogenic *E. coli* showed significantly lower survival rates compared to the wild-type (WT) strain of *E. coli*.^23^ Given its critical role in regulating bacterial virulence, targeting MiaA presents a promising avenue for novel antibiotic development. The third significant hit, PdxY, is known as a pyridoxal kinase involved in the pyridoxal 5ʹ-phosphate (PLP) salvage pathway. Its activity, however, is markedly lower compared to PdxK, suggesting that PdxY may play additional physiological roles beyond PLP salvage.^26,27^ The Cys121 residue of PdxY is involved in substrate-pyridoxal binding through covalent interaction.^28^

To confirm the interaction between 10-F05 and three potential target proteins identified through ABPP, we expressed and purified the recombinant *S. flexneri* FabH (Sf_FabH), Sf_MiaA and Sf_PdxY, proteins from *E. coli* BL21 strains. We then visualized the protein labeling with the cysteine-reactive tetramethylrhod-amine-5-iodoacetamide (5-TMRIA) fluorescent probe in competition with 10-F05. As expected, preincubation with 10-F05 resulted in a dose-dependent decrease in 5-TMRIA labeling of these proteins (Figure 3D), supporting that 10-F05 modifies their cysteine residues.

### Validation of FabH, MiaA, and PdxY as relevant targets for the bacterial inhibiting effects of 10-F05

**Figure 4.**
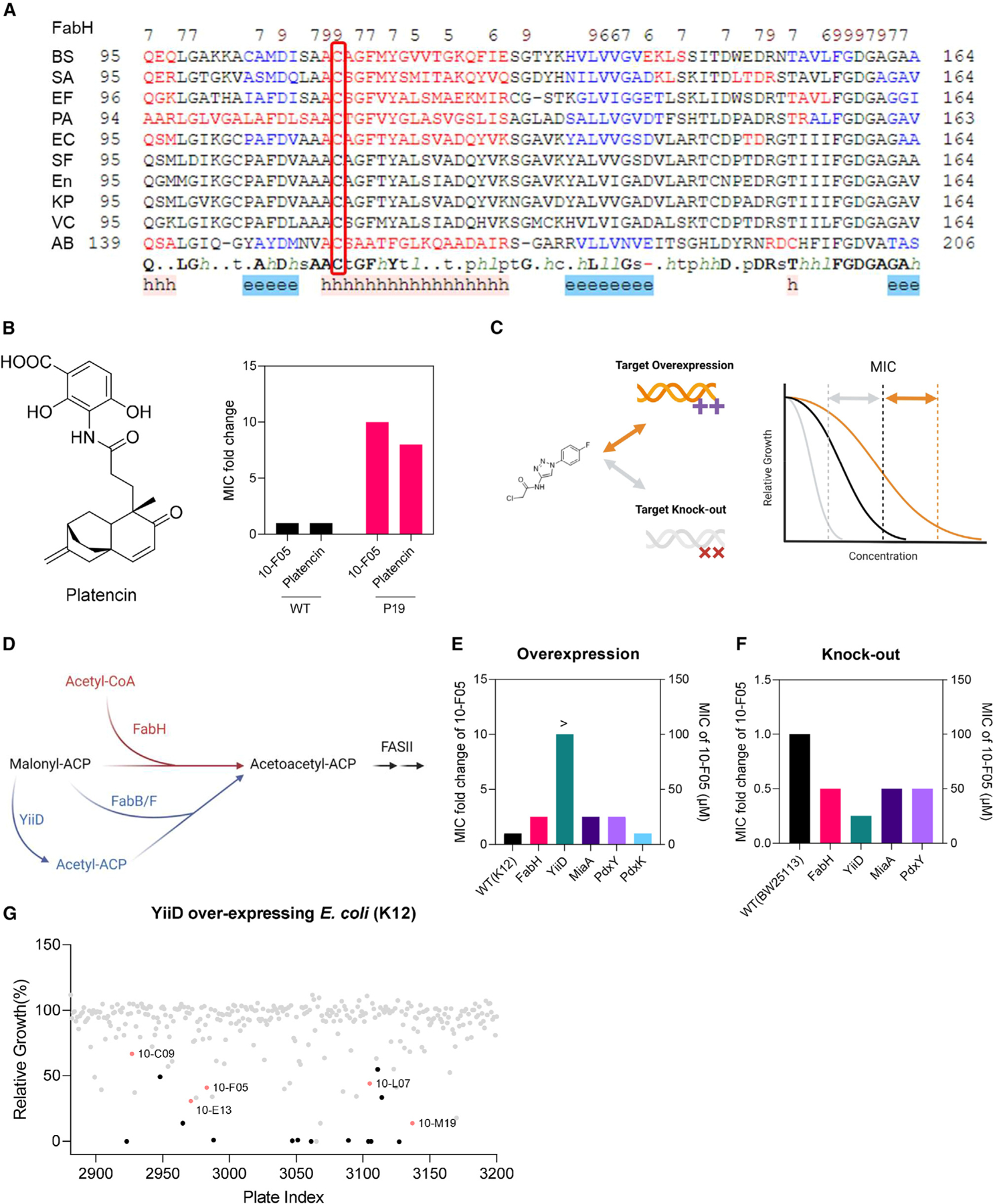
Chemogenetic validations suggested that FabH is the major target of 10-F05-mediated growth inhibition (A) Structural-based sequence alignment of FabH proteins from different bacteria species. BS, *B. subtilis* strain 168; SA, *S. aureus* MSSA476; EF, *E. faecium*; PA, *P. aeruginosa* ATCC 15692; EC, *E. coli* K12; SF, *S. flexneri* M90T; En, Enterobacter sp. (strain 638); KP, *K. pneumoniae* subsp. pneumoniae ATCC 700721; VC, *V. cholerae* serotype O1; AB, *A. baumannii* NCGM 237. The conserved cysteines are highlighted with a red box. (B) Left, structure of a reported FabH inhibitor, platencin. Right, MIC fold change of 10-F05 and platencin tested in WT and 10-F05-resistant *S. aureus* (passage 19 from Figure 2B). (C) Scheme for chemical genetic interaction. MIC shifts correspond to the target overexpression and knock-out. (D) YiiD bypasses FabH to generate acetoacetyl-ACP in *E. coli*. (E) MIC fold change of 10-F05 tested in target-overexpression *E. coli* strains (ASKA collection). (F) MIC fold change of 10-F05 tested in target knockout *E. coli* strains (Keio collection). >: No full growth inhibition was observed at the highest concentration tested, indicating that the real MIC was higher than the number reported here. (G) Plate 10 of Cys-library was rescreened in YiiD overexpressing *E. coli* (K12) strains at 25 µM. Triazole ring-conjugated chloromethyl ketone hits are labeled in red. Other hits are labeled in black.

Although 10-F05 targets all three proteins, further investigation is necessary to determine their contribution to its antibacterial activity. FabH is highly conserved across various bacteria (Figure 4A) and recognized as an antibiotic target. The *S. aureus* FabH, which shares 63% sequence similarity with Sf_FabH, can also be targeted by 10-F05 *in vitro* (Figure S5A). To assess the role of FabH inhibition in *S. aureus* by 10-F05, we utilized the known FabH inhibitor platencin (Figure 4B, left panel) and compared the MIC in a 10-F05-resistant *S. aureus* strain against a WT strain.^29^ The resistant *S. aureus* strain also showed resistance to platencin (Figure 4B, right panel), supporting the hypothesis that FabH inhibition is a key mechanism by which 10-F05 exerts its antibacterial effects in tested strains.

Further investigations were conducted using ASKA overexpression *E. coli* strains to confirm the importance of inhibiting FabH, MiaA, and PdxY in mediating the antibacterial activity of 10-F05 (Figure 4C), since the three target proteins are highly similar in *E. coli* K12 and *S. flexneri* M90T. Western blot analysis confirmed the overexpression of these proteins (Figure S5B).^30^ We then measured the MIC of 10-F05 in these strains, compared to the parental WT strain (Figure 4E). The expectation was that if a protein is a crucial target of 10-F05, its overexpression should result in an increased MIC. This was observed with FabH overexpression, which led to a 2.5-fold increase in MIC. Overexpression of YiiD (also known as FabY), which can compensate for the loss of FabH (Figure 4D),^20,31^ completely rescued the growth inhibitory effects of 10-F05. This difference can be explained by the fact that while overexpressed FabH is still targeted by 10-F05, YiiD is not inhibited by 10-F05. Overexpression of MiaA and PdxY similarly increased the MIC of 10-F05, reinforcing their relevance as targets. Notably, overexpressing PdxK did not affect the outcome of 10-F05 treatment, indicating a specific function for PdxY outside of pyridoxal salvage. Collectively, these findings underscore the role of these proteins as key targets of 10-F05.

To further demonstrate the covalent mechanism of 10-F05 in bacterial cells, we generated the BL21 *E. coli* strains overexpressing either WT or cysteine mutants (FabH_C112S, MiaA_C273A, and PdxY_C112A) and measured the MIC shift (Figure S5C). Since C112 of FabH and C121 of PdxY are known catalytic residues, the corresponding mutations rendered these enzymes catalytically inactive. Therefore, we did not expect to observe a loss of activity of 10-F05, which is confirmed in Figure S5C. C273 of MiaA is not involved in its enzymatic activity, and thus, we expected that the overexpression of MiaA C273 would significantly increase the MIC value of 10-F05. However, we did not observe a significant difference of 10-F05 MIC in BL21 strains overexpressing WT or C273A MiaA (Figure S5C). This is likely due to the minor effect of MiaA in supporting *E. coli* growth under normal LB media. We therefore further introduced the osmotic stress to increase the bacterial dependence on normal MiaA function (Figure 6D, middle).^23^ Under this condition, only the BL21 overexpressing C273A MiaA survived with 2.5 µM of 10-F05 treatment, while the strain overexpressing WT MiaA did not (Figure S5D). These findings support the covalent mechanism of 10-F05.

We further explored the impact of YiiD overexpression strain by re-evaluating the chloromethyl ketone compounds in the Cys-library. Notably, we observed that triazole-conjugated chloromethyl ketones lost their activity, while others retained activity, suggesting that the triazole-containing hits target FabH to produce the antibacterial effects while other hits may not rely on FabH targeting for their antibacterial activities (Figure 4G).

Additionally, we used knockout (KO) strains from the Keio collection^32^ to verify that FabH, MiaA, and PdxY are relevant targets of 10-F05. By comparing the MIC values of 10-F05 in WT and the *fabH/miaA/pdxY* KO strains, we discovered that the loss of any of these genes sensitized *E. coli* to 10-F05 treatment (Figure 4F). Notably, the *yiiD* KO strain showed the largest MIC change consistent with our overexpression data.

Since we obtained a 10-F05-resistant *S. aureus* P19, we sought to investigate the underlying resistance mechanism as a potential way to further confirm the targets of 10-F05. We first amplified and sequenced the *fabH* region by Sanger sequencing; however, no mutations were detected in the *fabH* sequence. This is probably not surprising as the 10-F05-targeted catalytic cysteine residue is essential for FabH activity. We then applied whole-genome sequencing to identify potential mutations associated with resistance (Table S7). A total of 25 high-confidence mutations were identified in the *S. aureus* P19. None of these mutations appeared to be directly related to pathways involving FabH-mediated fatty acid biosynthesis. Given the role of YiiD in bypassing FabH inhibition in *E. coli* and *S. flexneri* (Figure 4D), we hypothesized that the upregulation of a YiiD-like protein in *S. aureus* could be a potential mechanism of resistance. To explore this, we performed a BLAST search in MSSA476 and identified two potential YiiD-like proteins: SA_SAS0989 and SA_SAS1368. According to the UniProt, SAS0989 is annotated as a putative acetyltransferase, while SAS1368 is predicted to be involved in lipid transport or fatty acid metabolism. The transcriptional levels of FabH and SAS1368 showed no significant differences across three passages, with or without 25 µM 10-F05 treatment detected by qPCR (Figure S5E). However, 10-F05 treatment consistently upregulated the transcription of *SAS0989* across all three passages. Notably, the upregulation was significantly higher in the P19 *S. aureus* compared to the WT strain, suggesting a potential mechanism for bypassing FabH inhibition in 10-F05-resistant *S. aureus*.

Therefore, our integration of chemical proteomics with biochemical and genetic analyses revealed that multiple targets contribute to the observed antimicrobial activity of 10-F05, with FabH playing a major role.

### 10-F05 disrupts MiaA and tRNA substrate binding

**Figure 5.**
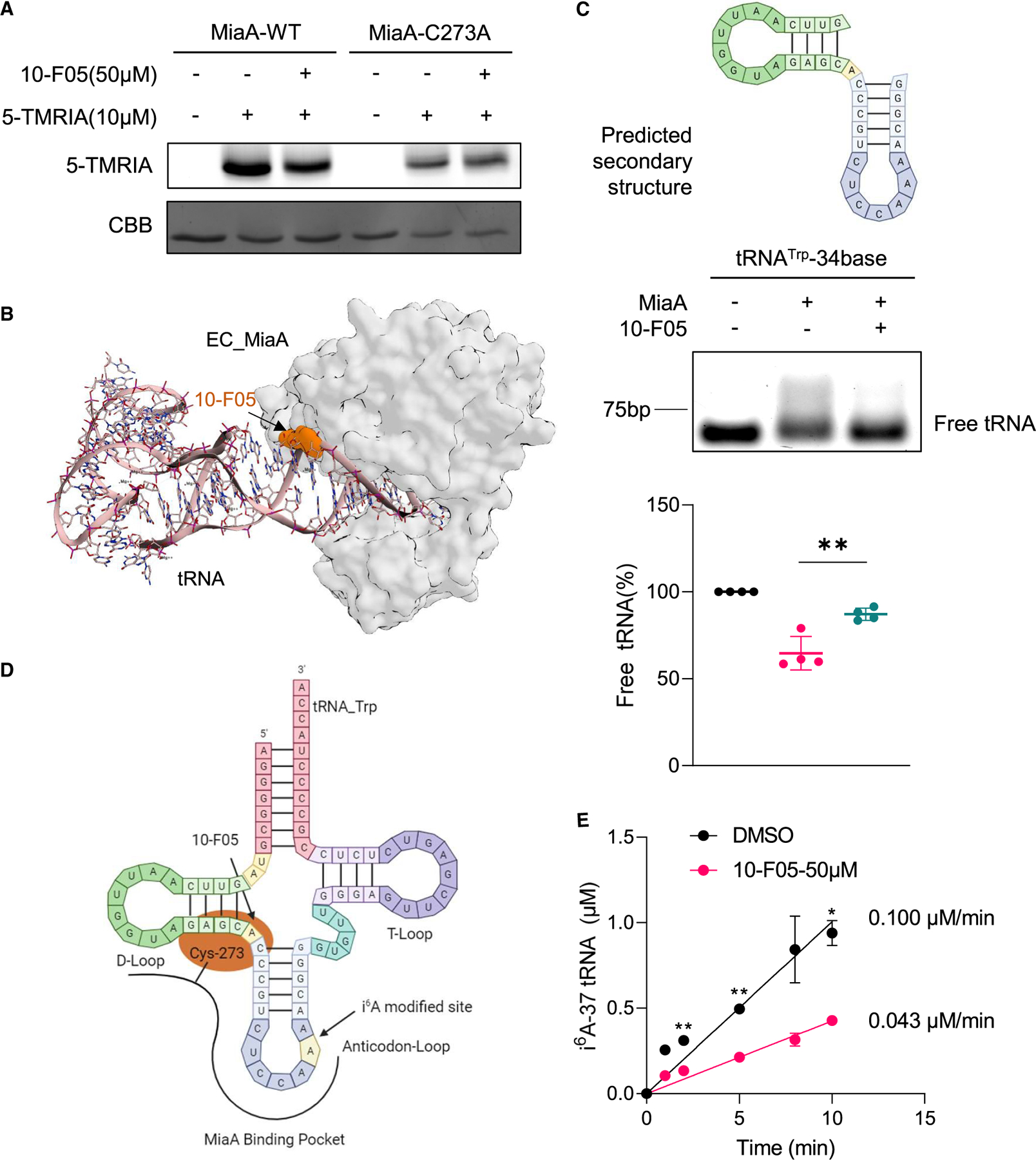
10-F05 disrupts MiaA and tRNA substrate binding (A) In-gel fluorescence of purified WT and C273A MiaA with and without 10-F05 as the competitor. Uncropped gel images are shown in supporting information. (B) Computational model of 10-F05 bound to *E. coli* MiaA (PDB: 2ZM5) by MOE (2020). 10-F05 is shown as orange surface. The 10-F05 binding site overlaps with the tRNA substrate-binding site. (C) Top: predicted secondary structure of designed tRNA sequence; middle: gel-based tRNA mobility shift assay; bottom: quantification of the ratio of unbound (free) tRNA (*n* = 4). (D) Simplified model showing that the linker region between anticodon loop and D-loop of tRNA is blocked by 10-F05 addition. (E) i^6^A-37 tRNA formation rate monitored by LC-MS in an *in vitro* enzymatic assay (*n* = 2). The *p* values of unpaired two-tailed t tests were calculated in GraphPad Prism (version 9.3.1). Data were plotted using mean ± SD, *p < 0.05, **p < 0.01.

Among the top three cysteine targets identified, the cysteines in FabH and PdxY play roles in catalysis. However, MiaA Cys273 is not a catalytic cysteine and is found only in a subset of bacterial strains (Figures S6A and S6B). Thus, we aimed to investigate whether covalent modification of MiaA Cys273 by 10-F05 affects MiaA’s function. Sf_MiaA contains a total of two cysteines. To confirm that 10-F05 reacts with MiaA Cys273, we purified the MiaA C273A mutant and performed 5-TMRIA labeling by in-gel fluorescence scanning. The labeling of the C273A was reduced, and this labeling was not affected by competing with 10-F05, indicating that 10-F05 specifically reacts with MiaA Cys273, consistent with our proteomic data (Figure 5A).

MiaA Cys273 is located near the tRNA-binding site (Figure S6C). We performed covalent docking to visualize the interaction between 10-F05 and MiaA (Figure S6D). The predicted covalent docking structure revealed a spatial overlap between 10-F05 and the tRNA substrate, suggesting that the covalent modification by 10-F05 could interfere with tRNA binding (Figure 5B, 10-F05 is shown in orange surface). To test this, we designed a 34-base tryptophan tRNA (tRNA^Trp^_34-base) sequence predicted to mimic the tRNA D-loop and anticodon loop^33^ and conducted a gel mobility shift assay (Figure 5C). We incubated purified MiaA protein with either 10-F05 or DMSO, and then added the tRNA substrate. Bound and unbound tRNA substrate were separated by electrophoresis and visualized with Gel-Red staining and quantified to assess the unbound tRNA ratio. The unbound tRNA^Trp^_34-base ratio increased from 64% to 87% after preincubation with 10-F05, in agreement with our hypothesis (Figures 5C and 5D). As expected, the tRNA binding of MiaA C273A mutant, which does not react with 10-F05, was largely unaffected by 10-F05 treatment (Figure S7A).

To further examine whether 10-F05’s blockade of the tRNA substrate could influence MiaA’s activity, we performed an *in vitro* enzymatic activity assay.^34^ The reaction kinetics were measured by monitoring the i^6^A-37 tRNA product using liquid chromatography-mass spectrometry (LC-MS). As expected, preincubation of 10-F05 with MiaA decreased the initial velocity of modified tRNA formation rate by 2.3-fold, using tRNA^Trp^_34-base as the substrate (0.100 vs. 0.043 µM/min, Figure 5E).

### 10-F05 treatment reduces translation fidelity and stress resistance through MiaA inhibition

**Figure 6.**
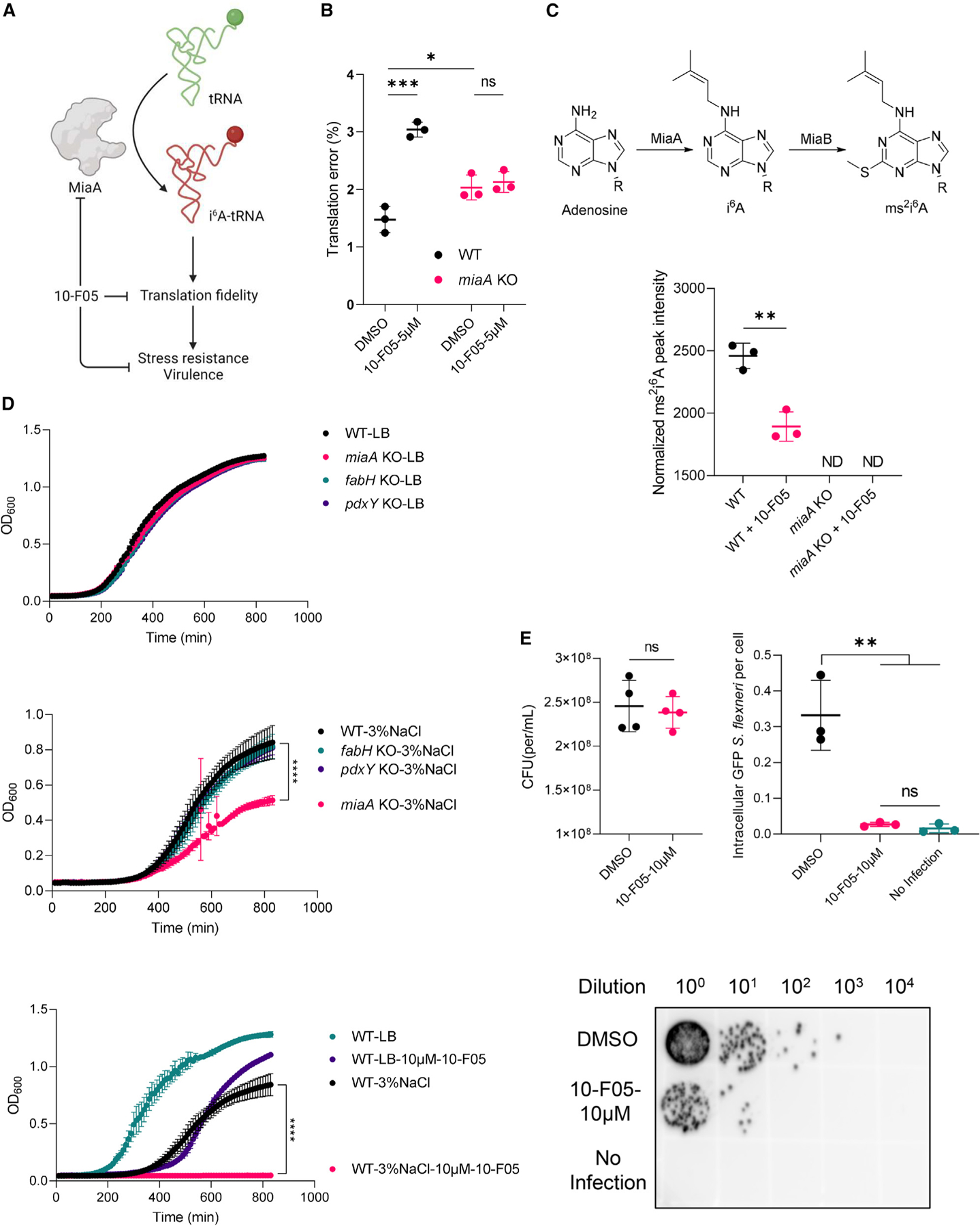
10-F05 increases translation error through MiaA inhibition *in vivo* to decrease bacterial stress resistance and virulence (A) Scheme showing MiaA’s role in regulating bacteria stress resistance and virulence. (B) Translation error measured using −1 frameshift reporter plasmids transformed in WT (BW25113) and *miaA* KO *E. coli* strains. (C) Top, biosynthesis pathway of i^6^A and ms^2^i^6^A tRNA. Bottom, normalized ms^2^i^6^A peak quantified by LC-MS. Corresponding tRNA samples were extracted from WT and miaA KO *E. coli* strains with or without 100 µM of 10-F05 treatment. ND: not detected (*n* = 3). (D) Top, growth curves of *E. coli* cultured in LB media. Middle, growth curves of *E. coli* cultured in 3% NaCl-LB media. Bottom, growth curve of WT *E. coli* cultured with or without 10 µM of 10-F05 in LB and 3% NaCl-LB media. (E) Top left, comparison of colony-forming unit (CFU) difference after 1-h treatment with 10 µM of 10-F05 (*n* = 4). Top right, comparison of intracellular GFP-*S. flexneri* level using Cytation 5 imaging. Intracellular GFP foci were quantified using ImageJ (1.53t). Three images of each group were acquired and analyzed. Bottom, intracellular *S. flexneri* level quantified using growth on agar plate. The *p* values of unpaired two-tailed t tests were performed in GraphPad Prism (version 9.3.1). Data were plotted using mean ± SD, *p < 0.05, **p < 0.01, ***p < 0.001, ****p < 0.0001, and ns, not significant.

We next investigated whether 10-F05 inhibits MiaA function in live bacteria (Figure 6A). Using a dual-luciferase reporter plasmid, we quantified the change in translation fidelity in the *miaA* WT and KO *E. coli* strains.^23^ We observed an elevated translation error in the *miaA* KO strain compared to the WT strain, confirming earlier findings.^23^ Further experiments involved treating both WT and *miaA* KO strains with 5 µM of 10-F05, revealing an increase in translation errors in the WT strain but not in the *miaA* KO strain. This observation strongly suggests that 10-F05 reduces translation fidelity by inhibiting the activity of MiaA (Figure 6B). Intriguingly, the measured translation error in 10-F05-treated WT *E. coli* cells exceeded that in *miaA* KO *E. coli* cells. These results likely suggest that *miaA* KO cells may have adapted to a lower translation error rate or due to other targets of 10-F05 that may contribute to translation error.

To further validate that 10-F05 inhibits MiaA in cells, we proceeded to purify the modified tRNA from *E. coli* and enzymatically digested it into individual ribonucleosides using established methods.^35,36^ Subsequently, we quantified the presence of i^6^A and ms^2^i^6^A (from i^6^A by the action of MiaB) using LC-MS analysis (Figure 6C, top panel). The i^6^A modification was barely detectable, falling below the sensitivity threshold of our LC-MS instrument. Nonetheless, we were able to detect a distinct peak corresponding to ms^2^i^6^A (same retention time as the synthetic standard of ms^2^i^6^A, Figures S7A and S7B). Importantly, this peak was only observed in the WT strain but not in the *miaA* KO strain, confirming the absence of i^6^A modification in the latter. As expected, when we treated the *E. coli* strain (starting from ~10^8^ highly concentrated inoculation for tRNA extraction) with 100 µM of 10-F05 for 3 h, we observed a ~24% decrease in the level of ms^2^i^6^A compared to the DMSO control group (Figure 6C, bottom panel). This ms^2^i^6^A reduction further supports 10-F05’s role in inhibiting MiaA activity in bacteria.

Previous studies on *S. flexneri* and *E. coli* have demonstrated that deleting *miaA* leads to a reduction in the expression of virulence factors and altered stress resistance (Figure 6A).^23,37,38^ In light of this, we tested if pharmacologically inhibiting MiaA with 10-F05 would produce similar outcomes. We observed that *fabH*, *miaA*, or *pdxY* KO strains did not significantly differ in growth from the WT strain when cultured in LB media (Figure 6D, top panel). However, under osmotic stress conditions (3% NaCl), the deletion of MiaA, but not the deletion of FabH or PdxY, resulted in a reduced *E. coli* growth rate (Figure 6D, middle panel). The addition of sub-MIC concentration of 10-F05 halted the growth of WT *E. coli* in 3% NaCl LB media but not in standard LB media, indicating that pharmacological inhibition of MiaA and possibly other proteins sensitized bacteria to osmotic stress (Figure 6D, bottom panel).

Building on previous findings that *miaA* deletion diminishes bacterial virulence, we examined if the MiaA inhibitor 10-F05 could similarly reduce virulence. We pre-treated GFP-tagged *S. flexneri* M90T (starting from ~3.2 × 10^8^ inoculation) with 10 µM of 10-F05 for 1 h. No growth inhibition was detected post 1-h treatment with 10 µM of 10-F05, as indicated by the colony-forming ability (Figure 6E, top-left panel). Subsequently, we infected HeLa cells with *S. flexneri* M90T cells, both with and without a 1-h pre-treatment of 10-F05. Given that *S. flexneri* is an intracellular pathogen whose pathogenesis relies on entering host cells, we evaluated changes in virulence by quantifying the number of intracellular bacteria. The average number of intracellular *S. flexneri* was significantly reduced with 10-F05 treatment (Figure 6E, top-right panel; also see Figure S7D). Similarly, an agar-based quantification demonstrated a substantial decrease in the intracellular levels of *S. flexneri*, indicating diminished virulence following 10-F05 treatment (>100-fold change, Figure 6E, bottom panel). Collectively, our findings indicate that 10-F05 treatment leads to a similar phenotype to *miaA* KO in bacterial pathogens, positioning MiaA as a viable target for controlling bacterial virulence and stress resistance.^23,37^

## DISCUSSION

By screening a library of ~3,200 cysteine-targeting compounds, we discovered a series of aromatic ring-conjugated chloromethyl ketone scaffolds exhibiting potent antibacterial activity. The lead compound, 10-F05, showed broad-spectrum activity against various bacteria and displayed a slow rate of resistance development. The competitive ABPP workflow enabled us to rapidly identify the molecular targets (FabH, MiaA, and PdxY) of 10-F05. Subsequent validation of their physiological significance in the growth inhibitory action of 10-F05 was achieved through chemical genetic interaction studies.

FabH has recently emerged as a promising target for antibiotic development. Various chemical scaffolds, including 1,3,5-oxadiazin-2-one,^19^ oxadiazolones,^4^ platencin and its derivatives,^21,29^ and benzoylaminobenzoic acid,^39^ have been reported as FabH inhibitors, acting through either covalent or non-covalent interactions. Our discovery of FabH as a target of 10-F05 strongly supports the efficacy of our integrated screening approach and ABPP platform in identifying valuable antibiotic targets. Notably, previously reported FabH inhibitors have low activity against gram-negative bacterial strains, primarily due to efficient drug efflux mechanisms.^22,39^ However, the electrophilic compound 10-F05, identified in our study, effectively inhibits FabH in gram-negative bacteria. This enhanced activity may be attributed to its smaller size, which possibly improves cell permeability, or to its rapid engagement with the target, potentially reducing drug excretion through the TolC efflux pump, thus maintaining its efficacy against gram-negative bacteria.

Until now, MiaA and PdxY have not been recognized as antibiotics targets, and to date, there have been no inhibitors reported that target them. This makes 10-F05 a promising foundation for the development of selective inhibitors for these new targets. Moreover, we validated that MiaA is a relevant antibacterial target for 10-F05. By examining published structures of MiaA and experimental validation, we discovered that the irreversible interaction between 10-F05 and MiaA Cys273 disrupts its interactions with the tRNA substrates. This interaction inhibits MiaA’s function, leading to reduced stress resistance and virulence in bacteria. The 10-F05 MIC changes observed in strains overexpressing PdxY and PdxK further suggest a previously unidentified role for PdxY, which appears to be unrelated to the PLP salvage pathway.

Initially, we were concerned about the promiscuity of 10-F05 due to its high reactivity. However, our proteomic results have shown that 10-F05 demonstrates reasonable target selectivity in live bacterial cells at 50 µM. Furthermore, a prior study has indicated that a compound’s promiscuity does not necessarily correlate with reactivity,^14^ suggesting that future investigations are needed to understand the mechanism of 10-F05’s target selectivity. We also note a potential advantage in 10-F05’s ability to target multiple bacterial proteins, as this multi-target mechanism may reduce the likelihood of bacteria developing resistance to 10-F05. This is in contrast to the bacteria’s response to other common antibiotics, such as methicillin (Figure 2). The multi-target strategy complicates the bacteria’s ability to accumulate mutations across all targeted proteins, potentially offering a more durable antibiotic solution.

We acknowledge that further optimization of 10-F05 is essential for its development into a viable clinical antibiotic candidate. Nevertheless, we emphasize the integrated approach of phenotypic screening of electrophilic compound libraries with ABPP platforms as a general and rapid method for identifying new targets and their corresponding lead compounds. We are optimistic that ongoing medicinal chemistry efforts will improve the drug-like attributes of 10-F05 and lead to the discovery of effective new antibiotics.

Traditional phenotypic screening with non-covalent compound libraries faces challenges in identifying the protein targets of promising hits. The integration of electrophilic compound libraries and ABPP platforms effectively addresses this challenge, offering a promising avenue for pioneering research in drug discovery and chemical biology. However, the size and molecular complexity of electrophilic compound libraries is significantly smaller compared to their non-covalent counterparts. Therefore, to unlock the full potential of phenotypic screening with electrophilic compound libraries, there is a crucial need to invest in the expansion of these libraries.

### Limitations of the study

Our study only screened against pathogenic bacteria in nutrient-rich laboratory media. Under these conditions, the number of essential genes required for bacterial survival is limited to approximately 300–600, with fewer than 10% predicted to be druggable.^40^ Screening covalent compound libraries under more physiologically relevant growth conditions could significantly enhance the likelihood of identifying new antibiotics and targets. The antibacterial compound identified here, 10-F05, has not been tested in animal infection models. Further optimization of 10-F05 may be needed for animal studies.

## RESOURCE AVAILABILITY

### Lead contact

Requests for additional information on data, resources, and reagents should be directed to and will be fulfilled by the lead contact, Hening Lin (hl379@cornell.edu).

### Materials availability

The reagents generated in this study will be made available subject to a completed Materials Transfer Agreement and import permits.

### Data and code availability

- All data are publicly available as of the date of publication and are listed on the key resources table. The raw mass spectrometry chemoproteomics data have been deposited to the ProteomeXchange Consortium through the PRIDE partner repository with the dataset identifier PXD058002.
- No code is generated in this paper.
- Any additional information required to reanalyze the data reported in this paper is available from the lead contact upon request.

## STAR★METHODS

### EXPERIMENTAL MODEL AND STUDY PARTICIPANT DETAILS

#### Microbe strains

*E. coli* BAA-2340, *A. baumannii* BAA-1790, *P. aeruginosa* BAA-2110, *K. pneumoniae* 700603, *K. pneumoniae* 13883, *S. aureus* MSSA476, *S. aureus* 43300, *E. cloacae* 13047, *E. coli* K12 were purchased directly from ATCC. *V. cholerae* SAD30 was a kind gift from Prof. Tobias Dörr from Cornell University. *S. flexneri* 5a M90T was a kind gift from Prof. Neal M. Alto from UT Southwestern. Enteropathogenic *E. coli* (EPEC) strain JPN15 (serotype O127:H6) was purchased from BEI Resources (NR-50517). *E. coli* strains from Keio collection (JW1077-fabH, JW1628-pdxY, JW3859-yiiD, JW4129-miaA, parent strain BW25113) were purchased from Horizon Discovery. *E. coli* strains from ASKA collection (JW1077-fabH, JW1078-fabD, JW4129-miaA, JW1628-pdxY, JW3859-yiiD, JW2411-pdxK, JW0658-miaB) were from National BioResource Project (https://resourcedb.nbrp.jp/top.jsp). Growth conditions were reported in STAR Methods.

#### Cell lines

Human HEK293T (ATCC: CRL-3216, female) and HeLa cells (ATCC: CCL-2, female) were cultured in DMEM (Invitrogen) media supplemented with 10% (v/v) heat-inactivated fetal bovine serum (FBS, Invitrogen). A549 (ATCC: CCL-185, male) cells were cultured in RPMI-1640 (Invitrogen) with 10% (v/v) heat-inactivated FBS.

### METHOD DETAILS

#### Reagents

Cysteine focused covalent library containing 3200 compounds was purchased from Enamine (CYS-3200-25-Y-20). Other chemicals used are DMAPP (Dimethylallyl Pyrophosphate, 63180 Cayman Chemical), N^6^-(∆^2^-isopentenyl) adenosine (i^6^A, 20522, Cayman Chemical), 5,5-dithio-bis-2-nitrobenzoic acid (DTNB, Ellman’s Reagent, ThermoFisher, 22582), Tetramethylrhodamine-5-Iodoacetamide Dihydroiodide (5-TMRIA, single isomer, ThermoFisher, T6006), 2-Methylthio-N-6-isopentenyladenosine (ms^2^i^6^A, sc-484230, Santa Cruz), 10-F05 (MFCD21602491, 1 click chemistry), 10-F05-N (115.316.629, Aurora), 10-L07-N (115.315.060, Aurora), 10-I09-N (ST-0015, Combi-blocks), 10-J03-N(ST-1288, Combi-blocks). All purchased reagents were used as received without further purification unless otherwise noted.

#### MIC mjeasurement

The minimum inhibitory concentrations (MICs) of compounds were measured using the broth microdilution method.^10^ One stab using 10 µL tip from bacteria glycerol stock was inoculated into 5 mL of LB supplemented with selection antibiotics (Kanamycin for Keio collections and chloramphenicol for ASKA collections), and the culture was incubated at 37°C with shaking overnight. The overnight culture was diluted 1:10000 (~5 × 10^5^ CFU/mL) and then dispensed in to a 96-well plate containing serial dilutions of indicated compounds in DMSO. The plate was incubated at 37°C for 16–20 h with shaking at 80 rpm and the OD_600_ was measured using Cytation 5 plate reader (BioTek). MIC was determined as the lowest concentration for which no bacterial growth was observed. LB was used as the growth media for all the bacteria strains unless otherwise mentioned. For *S. aureus* and *A. baumannii* strains, tryptic soy broth (TSB, BD, DF0370-17-3) was used as the growth media instead of LB. For *P. aeruginosa* and *K. pneumoniae* strains, nutrient broth (NB, RPI, N15100) was used as growth media. All MIC measurements were performed in at least biological duplicates.

#### Liquid-based antibacterial susceptibility screening

Bacterial overnight culture was diluted 1:10000 into 50 mL LB (TSB for *S. aureus*). The diluted culture was first dispensed into a 96-well plate (Costar, 96 well flat bottom, 3370) and then transferred (30 µL per well) into 384-well plates (Greiner, 384 well flat bottom, 781101) using epMotion 96 (Eppendorf). Cysteine focused library was diluted into desired stock concentration using DMSO and transferred into the 384-well plates using epMotion96 to a final concentration of 25 µM. The plates were incubated at 37°C for 16–20 h with shaking at 80 rpm and the OD600 was measured using Cytation 5 plate reader (BioTek). Bacteria relative growth was calculated by normalizing to the DMSO control group. Screening results are reported in Table S4.

#### Cloning, protein expression, and purification

*S. flexneri* FabH, PdxY, MiaA, MiaA_C273A, *S. aureus* FabH in the pET28a (+) vector with an N-terminal His-tag (cloned using the EcoRI and XhoI sites) were purchased from Twist Bioscience. The corresponding plasmid was transformed into BL21(DE3) chemical competent *E. coli* for expression. The transformed *E. coli* was then inoculated from an overnight culture into the 2L of LB supplemented with 50 µg/mL of Kanamycin. The cells were grown at 37°C for ~4 h with shaking at 200 rpm until the OD600 reached 0.6–0.8. IPTG (0.2 mM) was added to induce protein expression and the culture was incubated at 18°C overnight with shaking. Cells were harvested by centrifugation (8000 × g, 5 min, 4°C). Bacterial pellets were frozen at −80°C for future use. Bacterial pellets were resuspended in cold lysis buffer [50 mM Tris pH 8.0, 500 mM NaCl, 0.5 mg/mL Lysozyme (Thermo Scientific, 89833), 1 mM phenylmethylsulfonyl fluoride (PMSF, Thermo Scientific, 36978) in PBS, and Pierce universal nuclease] for 30 min on ice. Then the pellets were sonicated on ice for 2 min at 50% amplitude three times. Lysate was then clarified by centrifugation (30 000 × g, 60 min, 4°C). The supernatant was first loaded onto pre-equilibrated Ni-NTA resin (Qiagen, 30210), then washed with 50 mL cold wash buffer (50 mM Tris pH 8.0, 500 mM NaCl, 20 mM imidazole), and eluted with cold elution buffer (50 mM Tris pH 8.0, 500 mM NaCl, 200 mM imidazole). The resulting elution was then concentrated using a 10 kD MWCO Amicon filter and fractionated in ÄKTA pure FPLC system using a Superdex 75 gel filtration column pre-equilibrated with protein storage buffer (20 mM Tris, pH 8.0, 60 mM NaCl, 1 mM DTT, 10% glycerol). Fractions containing corresponding proteins were then pooled, flash-frozen, and stored at −80°C for future use. The protein concentration was measured using a Braford assay (Thermo Scientific, 23200).

#### Mammalian cytotoxicity measurement

Mammalian cytotoxicity measurement was performed using Cell Titer-Glo 2.0 Cell Viability Assay (Promega, G9243) according to the manufacturer protocol. In brief, mammalian cells were seeded into 384-well white plates (Greiner, 781080) and incubated at 37°C for one day. Compounds from the Cys-Library were dispensed into the assay plate using epMotion 96. The plates were then incubated at 37°C for two days. An equal volume of Cell Titer-Glo 2.0 reagent was added into each well of the assay plate using epMotion 96 and incubated at 22°C for 10 min, followed by luminescence measurement using a Cytation 5 plate reader (BioTek). HEK293T cytotoxicity results are reported in Table S4.

#### Reduced DTNB assay

Reduced DTNB assay was performed following a previously published method.^14^ In brief, a master mix of reduced DTNB was prepared by incubating DTNB (50 µM) with TCEP (200 µM) in DTNB reaction buffer (20 mM sodium phosphate, 150 mM NaCl, pH 7.4) for 5 min at 22°C. Reduced DTNB was then transferred into a 384-well plate (Greiner, flat bottom, 781101) using epMotion 96 (Eppendorf). Tested compounds (200 µM) were then dispensed into the assay plate, followed by continuous UV measurement at 412 nm at 37°C in Cytation 5 plate reader (BioTek). The background absorbance at 412 nm was measured under the same conditions without DTNB of each compound and subtracted from corresponding absorbance value. The concentration of remaining TNB^2−^ was calculated based on the absorbance values and normalized to the initial absorbance of each compound. Linear regression using Prism was performed to fit the rate for the first 45 min of measurements. Results are reported in Table S4.

#### Antibiotic resistance induction and frequency of resistance (FoR) measurement

Resistance induction was performed following a modified version of a previously published method.^4^ In brief, the overnight bacterial culture was diluted 1:10000 into fresh LB media (TSB for *S. aureus*) and the MICs of the compounds were detemined. Cultures from the wells with 0.25 × MIC of compounds were diluted 1:100 in fresh media and the MIC measurement was repeated. The fold changes in MIC were determined by dividing the daily MIC values by the MIC value on day 1. FoR was measured following a previously published method with slight modification.^41^ In brief, the overnight bacterial culture was diluted 1:10^3^ to 10-F05 containing agar plates (2X, 4X, 8X MIC) for drug resistant colony counting and 1:10^6^ to no-antibiotic agar plate for starting CFU counting.

#### Time-dependent killing assay

*S. flexneri* M90T overnight culture was diluted 1:10000 into fresh LB media. The starting inoculation (~10^5^ CFU/mL) was aliquoted into 15 mL tubes containing indicated concentrations of 10-F05. The bacterial culture was subsequently incubated at 37°C with shaking. Small portions of bacterial culture were collected at indicated time points. Serial dilutions were then plated on agar plates and incubated at 37°C overnight to determine the CFU. Experiments were performed in biological duplicates.

#### In-gel fluorescence analysis

Purified proteins (1.5 µM) were added into 40 µL of PBS with 10-F05 or DMSO at indicated concentrations. After 1 h incubation at 22°C or indicated incubation time for time-dependent labeling experiments, 5-TMRIA (10 µM) was added into the solution to label the cysteines. The reaction mixtures were incubated for another 1 h at 22°C and then quenched by adding 8 µL of 6x Laemmli buffer and analyzed by SDS-PAGE. Proteins labeled by 5-TMRIA were detected using Krypton scanning in ChemiDoc Imaging System (Bio-Rad) and protein loading was measured by Coomassie blue staining. Experiments were performed in biological duplicates and representative images were shown.

#### Cell culture and protein labeling for TMTpro18plex-based proteomics

In brief, overnight culture of indicated bacteria was harvested (4,500 × g, 10 min, 4°C), washed, and resuspended in 100 µL of PBS to give a final theoretical OD_600_ of 40. Then 50 µM of 10-F05, 10-L07, or DMSO (solvent control) were added into the live bacteria suspension and incubated at 37°C with shaking for 2 h. Bacterial cells were pelleted (4500 × g, 4°C, 10 min) and washed with PBS twice. Cells were resuspended in cold solution of DPBS containing Pierce Protease and Phosphatase Inhibitor Mini Tablet (ThermoFisher, A32961) or 100 µL Halt Protease and Phosphatase Inhibitor Cocktail (ThermoFisher, 78446) (1 tablet or 100 µL per 10 mL), then lysed using a Branson 550 probe sonicator (3 × 10 pulses, 0.4 s, 40% power, 4°C). Soluble fractions were then normalized to 2.0 mg mL^−1^ using the DC Protein Assay (BioRad) and absorbance was measured using a BioTek Cytation 5 plate reader following manufacturer’s instructions (BioTek Instruments, Winooski, VT, BTCYT5MPW). Normalized cellular lysates were then treated with desthiobiotin-tagged iodoacetamide probe^42^ (100 µM) at ambient temperature for 1 h by rotating end-over-end (20 rpm). Proteins were precipitated with 600 µL of cold methanol (−20°C), 200 µL of CHCl_3_ and 100 µL of chilled water (4°C). Following centrifugation (15,000 rpm, 10 min, 4°C), a protein disk formed at the interface of CHCl_3_ and aqueous layers. Both layers were aspirated without perturbing the disk, which was resuspended in cold methanol (600 µL, −20°C) and CHCl_3_ (200 µL, 4°C) by vortexing and sonicating using a sonicator equipped with a horn cup (1 × Qsonica Q700, Amplitude = 100, Process time = 20 s, Pulse-ON time = 2 s, Pulse-OFF time = 1 s, 4°C). The proteins were pelleted (15,000 rpm, 10 min, 4°C), and 100 µL of the digestion buffer (8 M urea, 50 mM TEAB, pH 8.5) was added to the resulting pellets. The pellets were resuspended with sonication and agitated on a thermal mixer (65°C, 10 min, 1,000 rpm). Then, 5 µL of 200 mM dithiothreitol in water was added to each sample, and the mixture was agitated on a thermal mixer (65°C, 10 min, 1,000 rpm). Next, 10 µL of 100 mM iodoacetamide in water was added to each sample, and the mixture was agitated on a thermal mixer (37°C, 30 min, 1,000 rpm).

#### Trypsin/lysine-C digestion and streptavidin enrichment

40 µg of Pierce Trypsin/Lysine-C Protease Mix (ThermoScientific, MS-Grade A40007) was reconstituted in 60 µL of 50 mM acetic acid and 20 µL of 100 mM calcium chloride. Samples were diluted with 400 µL of 50 mM TEAB (ThermoScientific, 90114) and 4 µL of the Trypsin/Lysine-C solution. Proteins were digested with agitation overnight on a thermal mixer (37°C, 1,000 rpm). To each sample was then added 500 µL of the enrichment buffer (50 mM TEAB, 0.2% IgepalTM CA-630, pH 8.5) containing 50 µL of Pierce Streptavidin agarose resin (ThermoFisher, 20353). Samples were enriched by rotating end-over-end (20 rpm) for 3 h at ambient temperature. Samples were next transferred onto Micro Bio-Spin Chromatography Columns (Bio-Rad, 7326204) and washed with the wash buffer (3 × 50 mM TEAB, 150 mM NaCl, 0.1% Igepal CA-630), DPBS (3×), and water (3×) by carefully aspirating from the bottom of each Bio-Spin column without drying the resin. Peptides were eluted with 50% acetonitrile in water containing 0.1% formic acid and each sample evaporated to dryness using vacuum centrifugation overnight (Savant, SpeedVac SPD-2030, temperature = 40°C, vacuum pressure = 5.1 Torr).

#### TMTpro-18plex labeling

Peptides were redissolved in 100 µL EPPS buffer (200 mM, pH 8.5) with 30% acetonitrile. TMT tags (10 µL per channel in acetonitrile, 20 µg µL^−1^) were added to the corresponding tubes and agitated on a thermal mixer (25°C, 90 min, 1,000 rpm). Each reaction was quenched by the addition of 10 µL of 5% hydroxylamine and mixed (25°C, 15 min, 1,000 rpm). To each sample, 10 µL of formic acid was added and mixed (25°C, 5 min, 1,000 rpm). TMT-labeled samples were combined into a single Protein LoBind microcentrifuge tube and evaporated to dryness using vacuum centrifugation.

#### Peptide desalting

Sep-Pak C18 cartridges (Waters, WAT054955) were conditioned with acetonitrile (3×) and desalting buffer (3×, 95% water, 5% acetonitrile, 0.5% formic acid). TMT-labeled peptides were redissolved in 500 µL of the desalting buffer, loaded dropwise onto the cartridge, and eluted at the rate of approximately 1 drop per second. The cartridge was then reloaded with the flow-through and subsequently desalted by slowly passing desalting buffer (3 × 1 mL). The peptides were eluted by adding 500 µL of 80% acetonitrile, 20% water, 0.5% formic acid (3×), eluates were combined into a clean Protein LoBind microcentrifuge tube, and sample was evaporated to dryness using vacuum centrifugation.

#### High pH reverse-phase fractionation

The spin columns for high pH fractionation (Pierce high pH reverse-phase peptide fractionation kit, ThermoScientific, 84868) were pre-equilibrated according to manufacturer’s instructions prior to use. Desalted peptides were redissolved in 0.1% trifluoracetic acid aqueous solution and loaded onto the column. The columns were spun down (2,000 × g, 2 min) with eluate retained, washed with 300 µL of water with eluate retained, and subjected to fractionation with a series of 0.1% triethylamine/acetonitrile buffers (2,000 × g, 2 min) with each eluate collected into a clean Protein LoBind tube. The following buffers were used for peptide elution (% acetonitrile): 5, 7, 9, 11, 12, 13, 14, 15, 16, 17, 18, 19, 20, 21, 22, 23, 24, 25, 26, 27, 28, 29, 30, 35, 40, 45, 50, 80. Fractions were evaporated to dryness using vacuum centrifugation, resuspended in water, and peptide concentrations determined using NanoDrop One Spectrophotometer (ThermoScientific, ND-ONEC-W, version 2.2.0.16). Fractions were combined with at least 7 fractions separation to yield 10 total fractions with approximately equivalent peptide amounts, filtered through CoStar Spin-X columns (Corning, 8160) and evaporated to dryness. The resulting 10 fractions were each reconstituted in 62 µL of 2% acetonitrile with 0.5% formic acid for subsequent nanoLC-MS/MS analysis.

#### Nano-scale reverse phase chromatography and tandem MS (nanoLC-MS/MS)

The nanoLC-MS/MS analysis was carried out using an Orbitrap Eclipse (ThermoScientific, San Jose, CA) mass spectrometer equipped with a nanospray Flex Ion Source coupled with the UltiMate 3000 RSLCnano (Dionex, Sunnyvale, CA). Each reconstituted fraction (3.5 mL = 0.7 µg for global proteomics fractions) was injected onto a PepMap C-18 RP nano trap column (5 µm, 100 µm × 20 mm, Dionex) at 20 µL min^−1^ flow rate for rapid sample loading, and separated on a PepMap C-18 RP nano column (2 µm, 75 µm × 25 cm). The column was equilibrated with 2% acetonitrile in 0.1% aqueous formic acid (eluant A) prior to each run. The labeled peptides were eluted in a 120-min gradient of 5%–33% eluant B containing 95% acetonitrile in 0.1% formic acid at 300 nL min^−1^, followed by an 8-min ramping to 90% B, a 7-min hold and 21-min re-equilibration with 2% acetonitrile and 0.1% formic acid prior to the next run. The Orbitrap Eclipse was operated in positive ion mode with nano spray voltage set at 1.9 kV and source temperature at 300°C. External calibration for FT, IT and quadrupole mass analyzers were performed. Raw MS data files for all the fractions were acquired using a real-time search (RTS) synchronous precursor selection (SPS) MS^3^ workflow as reported previously.^43^ Specifically, the RTS MS3 workflow consisted of 2.5 s “Top Speed” data-dependent CID-MS/MS scans (for peptide identifications by RTS) that enabled to trigger SPS of 10 MS2 product ions for subsequent MS3 in FT. In RTS node, the *Shigella flexneri serotype 5a* (strain M90T) FASTA database containing 3986 sequences was imported along with trypsin as the enzyme for real-time spectral database search for the samples from corresponding species. The search parameters included: TMTpro modification on N-terminal amines (∆ mass 304.2071) and carbamidomethyl modification of cysteine (∆ mass 57.0215) as static modifications; TMTpro modification (∆ mass 304.2071) on lysine, desthiobiotin-iodoacetamide probe on cysteine (∆ mass 296.1848), and methionine oxidation (∆ mass 15.9949) as dynamic modifications; maximum 3 variables per peptide; and 2 maximum missed cleavage allowed. A maximum search time of 35 ms was allowed for the RTS MS3 searching. The MS3 scan was carried out using a mass range of 110–500 m/z, an MS isolation window of 1.1 m/z and MS2 isolation window of 2.0 m/z were used. A resolving power of 50,000 at MS3 with a normalized collision energy of 55% was used for peptide quantitation. Other parameters included 200% normalized AGT target and 120 ms for maximum injection time. Dynamic exclusion parameters were set at 1 count within 50s exclusion duration with ±10 ppm exclusion mass window. All data were acquired under Xcalibur 4.4 operation software in Orbitrap Eclipse (ThermoScientific, San Jose, CA).

#### Data processing, protein identification, and data analysis

All raw MS spectra were processed and searched using the Sequest HT search engine within the Proteome Discoverer 3.0 (PD3.0, ThermoScientific). The same database for human proteins used for RTS data acquisition as described above was used for post-MS database searches. The default search settings used for 18-plex TMT quantitative processing and protein identification in PD3.0 searching software were: two mis-cleavage for full trypsin with fixed carbamidomethyl modification of cysteine, fixed 18-plex TMT modifications on lysine and N-terminal amines along with variable modifications of methionine oxidation, and protein N-terminal acetylation. The peptide mass tolerance and fragment mass tolerance values were 10 ppm for MS survey scan, 0.6 Da for MS2 and 20 ppm for MS3, respectively. Identified peptides were further filtered for maximum 1% FDR using the Percolator algorithm in PD3.0 along with additional peptide confidence set to high and peptide mass accuracy ≤5 ppm. The TMT18-plex quantification method within Proteome Discoverer 3.0 software was used to calculate the reporter ion abundances in MS3 spectra that were corrected for the isotopic impurities. Both unique and razor peptides were used for relative protein quantitation. Signal-to-noise (S/N) values were used to represent the reporter ion abundance with a co-isolation threshold of 50% and an average reporter S/N (intensity) threshold of ≥10 used for quantitation spectra. The intensities of peptides, which were summed from the intensities of the PSMs, were summed to represent the abundance of the proteins. For relative ratio between the two groups, normalization on sum of total peptide intensities for each sample was applied. The search results including ratio, peptide abundance for each sample were output to Microsoft Excel software for further data analysis. Processed results are reported in Table S6.

#### tRNA preparation

34-nucleotide RNA oligomers were purchased from Integrated DNA Technology (idtdna.com). The sequence used in the study is: tRNA^Trp^-34bases, [GUUCAAUUGGUAGAGCACCGGUCU-CCAAAACCGG]. RNA oligomers were reconstituted in tRNA folding buffer (30 mM HEPES, 100 mM KCl, 2 mM MgCl_2_, and 50 mM ammonium acetate pH 7.0). To ensure homogeneous folding of RNA into predicted structure with two hairpin loops, 50 µL of RNA solutions were heated up to 85°C over the course of 2 min and then cooled to 4°C over the course of 35 min in a PCR thermal cycler (Applied Biosystems Veriti 96-well Thermal Cycler). tRNA secondary structure prediction was performed as previously described.^44^

#### MiaA binding assay

MiaA binding assay was performed following a previously published method with modifications.^45^ Purified MiaA (10 µM) was incubated with 10-F05 (100 µM) or DMSO in 40 µL tRNA binding buffer (50 mM Tris-HCl pH 8.0, 100 mM NaCl, 10 mM MgCl_2_) for 1 h at 22°C. The folded tRNA (1 µM) was added into the mixture and incubated for another 20 min. The reaction mixtures were then supplemented with 8 µL of 6x Tri tracker loading dye (ThermoFisher) and separated on a 2% agarose TAE gel pre-stained with GelRed (Biotium, 41003) for 20 min at 100 V. Gels were then visualized in ChemiDoc Imaging System (Bio-Rad) and analyzed using ImageJ (1.53t). The unbound tRNA ratio was calculated by normalizing to the intensity of tRNA-only group. Experiments were performed in biological quadruplicates.

#### MiaA activity assay

MiaA activity assay was performed following a previously published method.^34^ Purified MiaA (200 nM) was incubated with 10-F05 (50 µM) or DMSO in 800 µL TMD buffer (60 mM Tris-HCl pH 7.5, 20 mM MgCl_2_, and 2 mM DTT) at 22°C for 1 h. Dimethyl allyl pyrophosphate (100 µM, DMAPP), bovine serum albumin (100 µg, BSA) and folded tRNA (6 µM) were then added to isopentenylate the A37 residue. Sample aliquots (60 µL) were removed at selected time points (0–10 min), heat-denatured at 95°C for 5 min. Nuclease P1 (M0660S, NEB, 200 units/mL in 30 mM sodium acetate buffer pH 5.4) and ZnSO_4_ (10 mM) were immediately added into the cooled aliquots and incubated with shaking for 16 h at 37°C. 10X CutSmart Buffer (NEB, B6004) and quick CIP (NEB, M0525S, 1 µL of 5000 units/mL) were added into the aliquots and incubated for 4 h at 37°C to yield the ribonucleosides. Proteins were removed by adding 50 µL of cold acetonitrile and centrifugation (17000 × g, 10 min, 22°C). Supernatants were collected for LC-MS analysis. The reaction components were eluted at a flow rate of 0.3 mL/min with the following time program: 0% B from 0 to 2 min, 0% B to 100% B from 2 to 5 min, 100% B from 5 to 10 min, 100% B to 0% B from 10 min to 12 min. Buffer A was 0.1% formic acid in HPLC-water and buffer B was 0.1% formic acid in HPLC-acetonitrile. The mass spectrometer was operated in positive ion mode. The observed m/z value of the [M + H] ^+^ stage of synthetic i^6^A standard (Cayman) was 336.2833 and the retention time was 5.27 min. Serial dilutions (0.01–10 µM) of synthetic standard i^6^A were prepared in 50% acetonitrile and 50% water and injected into LC-MS to generate the standard curve. Concentrations of i^6^A in each sample were then calculated based on the standard curve and the initial velocities were calculated using linear regression analysis in GraphPad Prism9. Experiments were performed in biological duplicates.

#### Molecular modeling

A reference MiaA structure from *E. coli* was downloaded from PDB database (2ZM5) and loaded into MOE (2020) software as Biomolecule Assembly with default settings. The protein structure was then prepared using the QuickPrep function with the default parameters and thoroughly checked using the Structure Preparation function. The tRNA was first removed from the binding pocket. The binding site on MiaA was defined based on the identified cysteine position. 10-F05 was docked onto Cys273 using Covalent Docking (Reaction: alpha-halocarbonyl, thioether; Refinement: Induced fit). The resulting docking pose was then superposed with the tRNA-bound MiaA structure and visualized in MOE and PyMol (4.6.0).

#### Frameshift quantification

Frame shift quantification was performed as previously described.^23^ The plasmids we used are kind gifts from Dr. Matthew A. Mulvey. Briefly, *E. coli* WT and *miaA* KO strains (Keio collection) transformed with pCWR44 and pCWR45 were grown overnight in LB supplemented with chloramphenicol for WT strains and both chloramphenicol (25 µg/mL) and kanamycin (50 µg/mL) for *miaA* KO strains. The cells were sub-cultured into fresh LB to give an OD_600_ of 0.1 at the day of experiment. 5 µM of 10-F05 was added into the bacterial culture and incubated at 37°C with shaking. After 30 min, arabinose (0.2%) was added into the bacterial cultures to induce the expression of luciferase. Bacterial cells were then pelleted when the OD_600_ reached 0.5 and resuspended in Passive Lysis Buffer (Promega, E1910), followed by mechanical lysis using disruption beads in TissueLyser LT (QIAGEN). Luciferase activities were analyzed using the dual luciferase reporter assay following the manufacturer protocol (Promega, E1910). Experiments were performed in biological triplicates. Translation error was calculated based on the following formula:

$$Translation\;error\left(\%\right)=\frac{LumFirefly\left(pCWR44\right)}{LumRenilla\left(pCWR44\right)}÷\frac{LumFirefly\left(pCWR45\right)}{LumRenilla\left(pCWR45\right)}\times 100\left(\%\right)$$

#### Quantification of ms^2^i^6^A-tRNA modification

tRNA purification was performed following a previously published method.^36^ In brief, bacterial overnight culture (WT and *miaA* KO *E. coli* strains) was diluted 1:10 into fresh media (25 mL) containing 100 µM of 10-F05 or DMSO as control. After 4 h incubation at 37°C with shaking, the bacterial cells were pelleted by centrifugation (4500 × g, 25 min, 4°C). The pellets were washed with 0.9% NaCl once and stored at −80°C for future use. Total nucleic acids were extracted from the pellet using 900 µL of extraction buffer (50 mM NaOAc, 10 mM MgOAc, pH 5.0) and 860 µL of acidic phenol pH 4.5 (Sigma, P4682) for 30 min at 37°C with shaking. The aqueous phases were collected after centrifugation (4500 × g, 15 min, 4°C). Next, 700 µL of extraction buffer was added into the phenol phase and the same procedures were repeated. The aqueous phases were combined. 75 µL of 5 M NaCl and 1.5 mL of isopropanol was added into the combined aqueous phases to precipitate the total nucleic acids by centrifugation (14500 × g, 15 min, 22°C). The pellets were washed with cold 70% ethanol and air dried for 10 min rRNA was removed by resuspending the pellets into 750 µL of cold 1 M NaCl and centrifugation (9500 × g, 20 min, 4°C). Remaining nucleic acids (DNA and tRNA) were precipitated by adding 1.5 mL of cold ethanol to the supernatants and incubated at −20°C for 30 min, followed by centrifugation (14500 × g, 5 min, 4°C). The pellets were washed with 70% cold ethanol and air dried for 10 min. DNA was removed by dissolving the pellets into 300 µL of 0.3 M NaOAc, pH 5.0 and precipitated by adding 170 µL of isopropanol, followed by incubation at 22°C for 10 min. The supernatants containing tRNA were collected after centrifugation (14500 × g, 5 min, 22°C). Next, 115 µL of isopropanol was added into the supernatant and incubated at −20°C for 30 min. The total tRNA was then prepared by centrifugation (14500 × g, 15 min, 4°C) and washed with 70% ethanol. A total of 25 µL DEPC-water was added to dissolve the tRNA pellet for each sample. The concentration and quality of tRNA samples were determined by Nanodrop.

Quantification of ms^2^i^6^A-tRNA modification was performed using LC-MS following a previously published method.^35^ In brief, approximately 12.5 mg tRNA samples were enzymatically digested into nucleosides in a total of 50 µL of digestion buffer [1 µL of Pierce Universal Nuclease (ThermoFisher, 88700), 0.5 µL of Nuclease P1 (NEB, M0600S, 5000 units/mL), 6 µL of Nuclease P1 buffer and 1 µL of Alkaline phosphatase (Sigma, P5521, 1000 units/mL)] and incubated at 37°C with shaking overnight. The reaction components were eluted at a rate of 0.3 mL/min with the following time program: 0% B from 0 to 2 min, 0% B to 100% B from 2 to 5 min, 100% B from 5 to 10 min, 100% B to 0% B from 10 min to 12 min. Buffer A is 0.1% formic acid in HPLC-water and buffer B is 0.1% formic acid in HPLC-acetonitrile. The mass spectrometer was operated in positive ion mode. The observed m/z value of the [M + H] ^+^ stage of synthetic ms^2^i^6^A standard (Santa Cruz) was 382.2901 and the retention time was 5.65 min. The normalized ms^2^i^6^A peak intensity was calculated by dividing the absolute intensity of the ms^2^i^6^A ions to the tRNA concentration of each sample measured by Nanodrop. Experiments were performed in biological triplicates.

#### Osmotic stress resistance assays

Bacterial overnight culture was diluted 1:1000 into 3% NaCl-LB or LB as indicated. 200 µL of diluted cultures were then dispensed into 96 well plates with 10 µM of 10-F05 or DMSO (control). OD_600_ curve was monitored using a Cytation5 plate reader every 10 min at 37°C with consistent shaking. Experiments were performed in biological triplicates.

#### Infection of mammalian cells with GFP-tagged S. flexneri M90T

GFP-tagged *S. flexneri* infection was performed as previously described with modifications.^46^ In brief, ~10^5^ HeLa cells were seeded into 24-well plates. An overnight culture of GFP-tagged *S. flexneri* M90T was diluted 100-fold in fresh LB and incubated at 37°C with shaking until the OD_600_ reached 0.4. The bacterial cells were then pelleted and resuspended in the same volume of PBS. The bacterial suspensions were treated with 10 µM of 10-F05 or DMSO for 1 h at 37°C with shaking. A small portion of the mixture was plated on LB agar plates overnight to count the CFU after drug treatment. 25 µL of bacterial suspensions were added into HeLa cells for an MOI of ~25. After 2 h incubation at 37°C, the HeLa cells were washed with PBS twice and incubated in fresh media containing 50 µg/mL gentamycin and Hoechst statin (Invitrogen, 33342) for 30 min. HeLa cells were then washed with PBS twice and imaged using Cytation5. Representative images are shown in Figure S7D. For the agar plates counting assay, HeLa cells were directly collected after 30 min incubation and lysed using 0.5% Triton X-100 PBS lysis buffer to release the intracellular bacteria. Serial dilutions from the lysates were plated on agar plates using spot format and incubated at 37°C overnight. Experiments were performed in biological duplicates.

#### Structural based sequence alignment

Protein structures were downloaded from Uniprot Database using either reported structures or AlphaFold2 predicted structures. Structure based sequence alignment was performed using PROMALS3D (http://prodata.swmed.edu/promals3d/promals3d.php).^47^

#### Quantitative reverse transcription PCR (RT-qPCR), PCR and whole genome sequencing for resistance investigation

*S. aureus* overnight culture was diluted 1:5 into fresh TSB containing either DMSO or 10-F05 for 2 h with shaking at 37°C. Bacteria pellets were then collected and washed with PBS twice for total RNA extraction using the E.Z.N.A. Bacterial RNA Kit (R6950–01). cDNA was synthesized using the MultiScribe Reverse Transcription Kit (4311235). Quantitative PCR was performed using 2x Universal SYBR Green Fast qPCR mix (ABclonal no. RK21203) by QuantStudio 7 Flex real-time PCR system. Primers used for RT-qPCR are listed in Supplementary Data Table S3. Primers used for amplifying target genome regions are also listed in Supplementary Data Table S3. The whole genome sequencing and data analysis was performed using Genewiz Short-Read Non-Human WGS service for P19 MSSA476.

#### Synthesis of 10-F05-CA

Chloroacetyl chloride (0.75 equiv, 0.375 mmol, 30 µL) was dissolved in 10 mL of dichloromethane (DCM) and cooled to 0°C. To this solution, 1-(4-fluorophenyl)-1H-1,2,3-triazol-4-amine hydrochloride (1.0 equiv, 0.5 mmol, 107 mg) and triethylamine (2.0 equiv, 1.0 mmol, 140 µL) were added. The reaction mixture was stirred at room temperature overnight. Upon completion, as monitored by TLC, the solvent was removed under reduced pressure. The crude product was purified by flash column chromatography using a 1:1 mixture of ethyl acetate and hexane, a white solid product 10-F05-CA was obtained with 89% of yield.

Synthesis was carried out following the scheme given below:


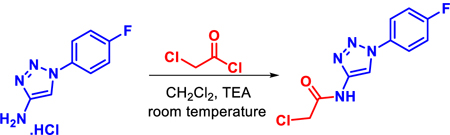


#### 2-Chloro-*N*-(1-(4-fluorophenyl)-1H-1,2,3-triazol-4-yl)acetamide (10-F05-CA)

**Physical State**: White Solid; Yield: 89%. ^**1**^**H NMR (400 MHz, DMSO)** δ 11.47 (s, 1H), 8.78 (s, 1H), 8.05–7.95 (m, 2H), 7.48–7.38 (m, 2H), 4.34 (s, 2H). ^**13**^**C NMR (101 MHz, DMSO)** δ 164.39, 162.2 (d, *J* = 246 Hz, C-F), 144.25, 133.69 (d, *J* = 2.98 Hz, C-F), 122.88 (d, *J* = 8.79 Hz, C-F), 117.12 (d, *J* = 23.28, C-F), 112.63, 42.94. **LCMS** (Thermo Fisher Scientific) m/z: [M + H] ^+^ Calcd for C_10_H_9_ClFN_4_O:255.2; Found 255.1.

### QUANTIFICATION AND STATISTICAL ANALYSIS

ImageJ was used to count the colonies on agar plates for CFU calculation and count the total cell number and GFP-tagged S. flexneri in the infection assay. GraphPad Prism9 was used for all the statistical analysis. Significance was calculated using Student’s t test or multiple comparisons. In the figures, * indicates *p* < 0.05, ** indicates *p* < 0.01, *** indicates *p* < 0.001, and **** indicates *p* < 0.0001. The number of biological replicates (n) is reported in the method part of STAR Methods details and figure legends. The images presented are representative of three independent biological replicates. Data are presented as mean ± standard deviation (SD). Statistical test methods were reported in figure legends.

## Supplementary Material

MMC7

MMC4

MMC1

MMC5

MMC2

MMC3

MMC6

## Figures and Tables

**Table:** KEY RESOURCES TABLE

REAGENT or RESOURCE	SOURCE	IDENTIFIER
Bacterial and virus strains		
*E. coli* BAA-2340	ATCC	BAA-2340
*A. baumannii* BAA-1790	ATCC	BAA-1790
*P. aeruginosa* BAA-2110	ATCC	BAA-2110
*K. pneumoniae* 700603	ATCC	700603
*K. pneumoniae* 13883	ATCC	13883
*S. aureus* MSSA476	ATCC	BAA-1721
*S. aureus* 43300	ATCC	43300
*E. cloacae* 13047	ATCC	13047
*E. coli* K12	ATCC	10798
*V. cholerae* SAD30	Gift from Prof. Tobias Dörr	N/A
*S. flexneri* 5a M90T	Gift from Prof. Neal M. Alto	N/A
Enteropathogenic *E. coli* (EPEC) strain JPN15	BEI Resources	NR-50517
*E. coli* strains from Keio collection	Horizon Discovery	JW1077-fabH
*E. coli* strains from Keio collection	Horizon Discovery	JW1628-pdxY
*E. coli* strains from Keio collection	Horizon Discovery	JW3859-yiiD
*E. coli* strains from Keio collection	Horizon Discovery	JW4129-miaA
*E. coli* strains from Keio collection	Horizon Discovery	BW25113
*E. coli* strains from ASKA collection	National BioResource Project	JW1077-fabH
*E. coli* strains from ASKA collection	National BioResource Project	JW1078-fabD
*E. coli* strains from ASKA collection	National BioResource Project	JW4129-miaA
*E. coli* strains from ASKA collection	National BioResource Project	JW1628-pdxY
*E. coli* strains from ASKA collection	National BioResource Project	JW3859-yiiD
*E. coli* strains from ASKA collection	National BioResource Project	JW2411-pdxK
*E. coli* strains from ASKA collection	National BioResource Project	JW0658-miaB
BL21(DE3)	NEB	C25271
Chemicals, peptides, and recombinant proteins		
Cysteine focused covalent library	Enamine	N/A
Dimethylallyl Pyrophosphate	Cayman Chemical	63180; CAS: 358-72-5
N6-(∆2-isopentenyl) adenosine	Cayman Chemical	20522; CAS: 7724-76-7
5,5-dithio-bis-2-nitrobenzoic acid	ThermoFisher	22582; CAS: 69-78-3
Tetramethylrhodamine-5-lodoacetamide Dihydroiodide	ThermoFisher	T6006; CAS: 114458-99-0
2-Methylthio-N-6-isopentenyladenosine	Santa Cruz	sc-484230; CAS: 20859-00-1
10-F05	1 click chemistry	MFCD21602491; CAS: 1375471-68-3
10-F05-N	Aurora	115.316.629; CAS: 1779798-64-9
10-L07-N	Aurora	115.315.060; CAS: 1781280-38-3
10-I09-N	Combi-blocks	ST-0015; CAS: 122-62-9
10-J03-N	Combi-blocks	ST-1288; CAS: 87674-21-3
Kanamycin Monosulfate	Gold Bio	K-120-5
Ampicillin (Sodium)	GoldBio	A-301-5
Chloramphenicol	Gold Bio	C-105-5
Tetracycline Hydrochloride	Gold Bio	T-101-25
Gentamicin Sulfate	GoldBio	G-400-1
Streptomycin Sulfate	GoldBio	S-150-50
Imipenem	GoldBio	I-600-1
Methicillin Sodium	GoldBio	M-830-1
Tryptic soy broth	BD	DF0370-17-3
Nutrient broth	RPI	N15100
Luria-Bertani Broth	VWR	90000-808
Lysozyme	Thermo Scientific	89833
Phenylmethylsulfonyl fluoride	Thermo Scientific	36978
Pierce universal nuclease	Thermo Scientific	88700
Ni-NTA resin	Qiagen	30210
GelRed	Biotium	41003
Nuclease P1	NEB	M0600S
Alkaline phosphatase	Sigma	P5521
Critical commercial assays		
Cell Titer-Glo 2.0 Cell Viability Assay	Promega	G9243
Dual luciferase reporter assay	Promega	E1910
Ε.Ζ.Ν.Α. Bacterial RNA Kit	Omega	R6950-01
MultiScribe^™^ Reverse Transcription Kit	Thermo Scientific	4311235
SYBR Green Fast qPCR mix	ABclonal	RK21203
Deposited data		
Competitive activity-based protein profiling mass spectrometry for 10-F05 and 10-L07 target identification	This paper	PXD058002
Experimental models: Cell lines		
Human: HEK293T	ATCC	CRL-3216
Human: Hela	ATCC	CCL-2
Human: A549	ATCC	CCL-185
Oligonucleotides		
tRNA^Trp^-34bases GUUCAAUUGGUAGAGCACCGGUCU-CCAAAACCGG	This paper	N/A
Primers for mutagenesis and qPCR, see supplementary Data	This paper	N/A
Recombinant DNA		
pET28a(+)-His-SF-FabH	Twist Bioscience	N/A
pET28a(+)-His-SF-PdxY	Twist Bioscience	N/A
pET28a(+)-His-SF-MiaA	Twist Bioscience	N/A
pET28a(+)-His-SF-MiaA_C273A	Twist Bioscience	N/A
pET28a(+)-His-SA-FabH	Twist Bioscience	N/A
pCWR44	Gift from Dr. Matthew A. Mulvey	N/A
pCWR45	Gift from Dr. Matthew A. Mulvey	N/A
Software and algorithms		
Graphpad Prism 10	Graphpad	https://www.graphpad.com
MOE2020	Chemical Computing Group	https://www.chemcomp.com/en/Products.htm
PyMol	PyMol	https://www.pymol.org/
Other		
Antibacterial Screening Result for the Cys-library, see supplementary Data	This paper	N/A
Cytotoxicity Screening Result for the Cys-library, see supplementary Data	This paper	N/A
DTNB Reactivity for the Cys-library, see supplementary Data	This paper	N/A

**Table 1.** MIC values of hit fragments bearing a chloromethyl ketone warhead

					MIC (µM)			
Core structure	Compound name	R_1_	R_2_	X	*SF*	*VC*	*EC*	*SA*
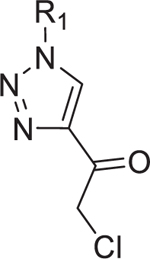	10-F05	C_6_H_4_(p)F	-	-	2.5	10	25	10
	10-L07	C_6_H_4_(m)CH_3_	-	-	5	25	50	10
	10-C09	C_6_H_4_(m)CF_3_	-	-	25	100	100	25
	10-M19	CH_3_	-	-	50	50	50	100
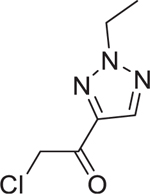	10-E13	-	-	-	25	50	100	50
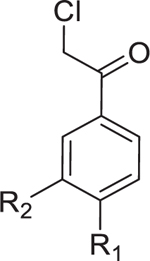	10-D10	OCH_3_	CI	-	10	25	100	25
	10-E07	H	SCH_3_	-	10	25	100	25
	10-F10	CN	H	-	5	10	25	10
	10-I13	SO_3_CH_3_	H	-	10	25	50	50
	10-L13	F	F	-	25	25	50	50
	10-L16	CI	CI	-	10	10	25	25
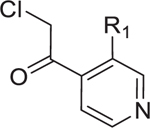	10-I09	F	-	-	50	10	50	50
	10-M09	H	-	-	25	25	25	50
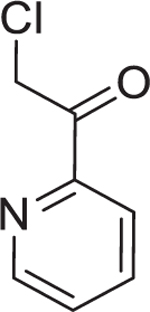	10-J03	-	-	-	50	10	100	25
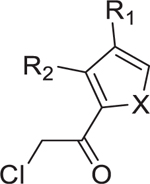	10-C05	CONH_2_	H	O	25	50	50	50
	10-L06	CONH_2_	H	S	25	50	50	25
	10-K11	H	CI	S	25	25	50	25
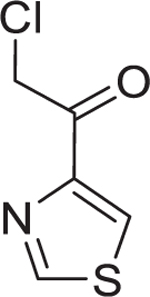	10-L08	-	-	-	25	25	25	25

## Highlights

- Integrated phenotypic screening and ABPP are applied for antibiotic target discovery
- 10-F05 is effective in several ESKAPE pathogens with slow resistance development rate
- 10-F05 targets FabH and MiaA to inhibit bacterial growth, stress resistance, and virulence

## SIGNIFICANCE

Screening small-molecule libraries for compounds with useful biological activities has been an important research endeavor in chemical biology. Most efforts in this direction involve screening of non-covalent compounds. Due to difficulty in target identification, screening of non-covalent compound libraries is more popularly done for specific target proteins, instead of cell-based phenotypic screening. Here, by screening for covalent inhibitors of bacterial growth, we demonstrate the unique capability of combined phenotypic screening of covalent compound library with activity-based profiling for target identification. This approach not only produces lead compounds but also identifies new druggable targets. We believe that cell-based phenotypic screening of covalent compound libraries could be broadly useful to the chemical biology community.
