# Supplementary material for: Antibiotic target discovery by integrated phenotypic and activity-based profiling of electrophilic fragments
Source: Cell Chem Biol. Author manuscript; Available in PMC 2025 Apr 14. (PMC11995724; doi:10.1016/j.chembiol.2025.02.001)
Supplement: MMC7 [file NIHMS2061764-supplement-MMC7.pdf]

Data S2. Uncropped gels. Related to Figure 3, Figure 5 and STAR methods.

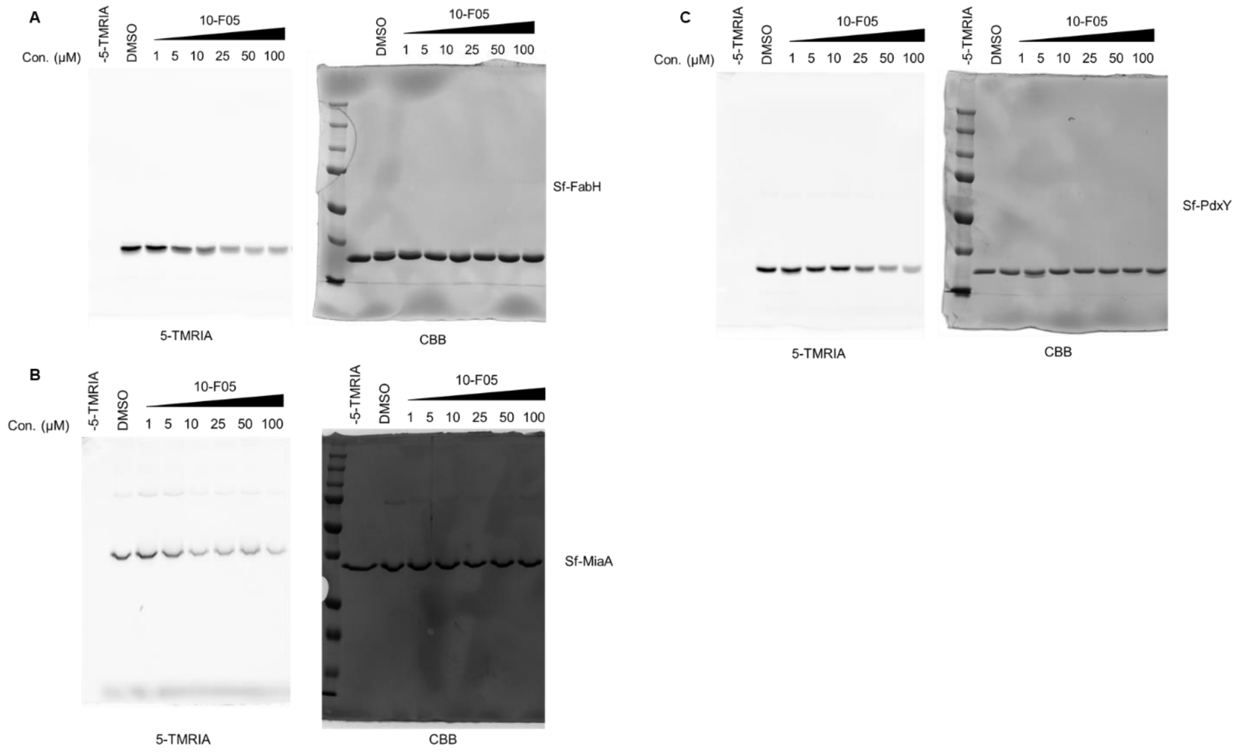

Uncropped gel for Figure 3D. Related to Figure 3.

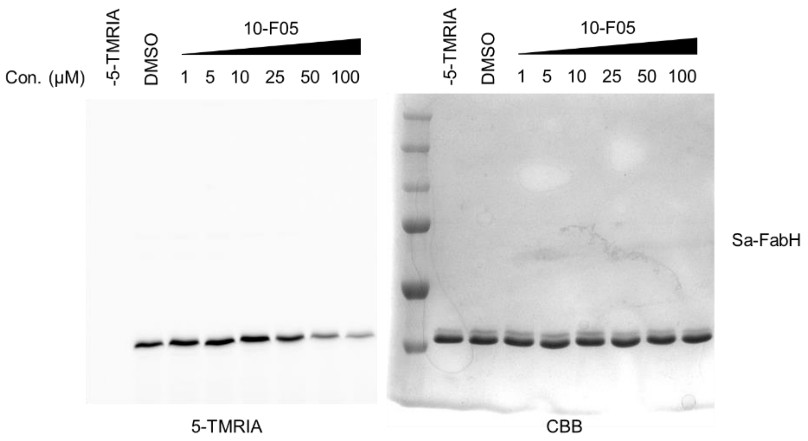

Uncropped gel for Figure S12. Related to STAR methods.

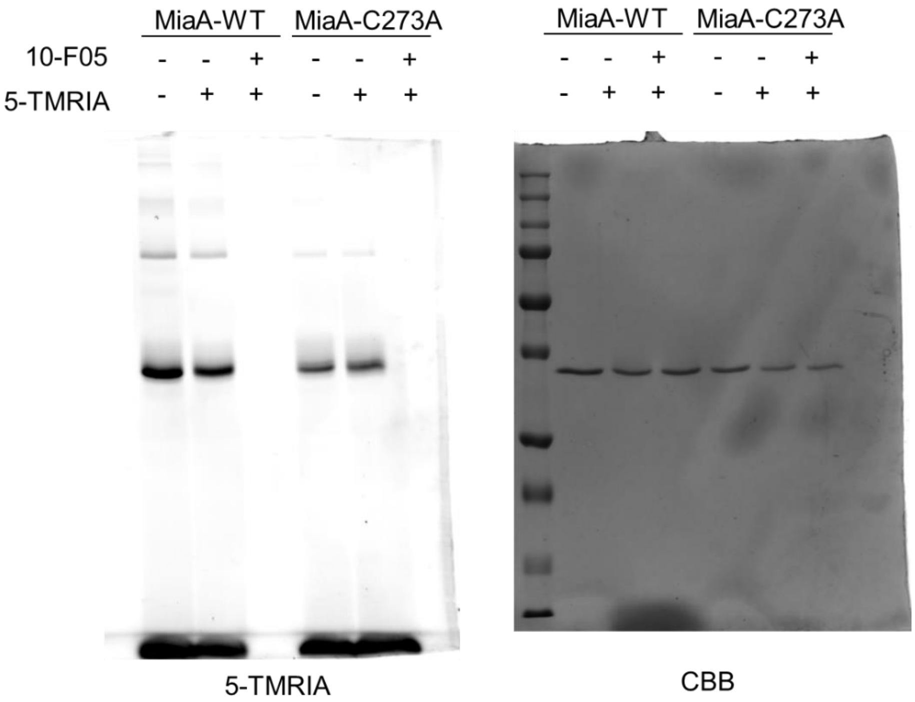

Uncropped gel for Figure 5A. Related to Figure 5.

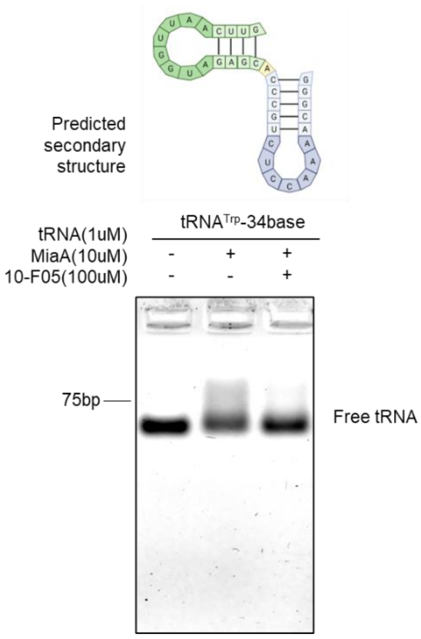

Uncropped gel for Figure 5C. Related to Figure 5.

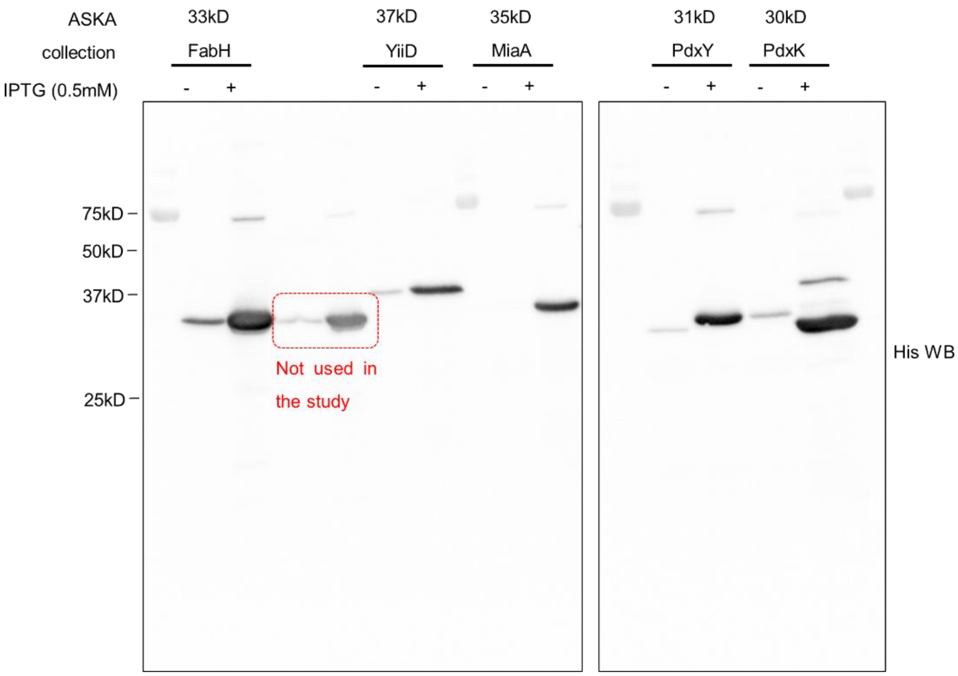

Uncropped blot for Figure S13. Related to STAR methods.

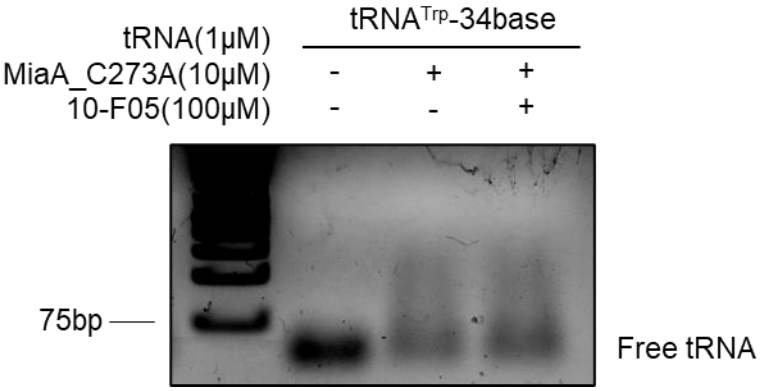

Uncropped gel for Figure S20. Related to STAR methods.
