# Supplementary material for: Antibiotic target discovery by integrated phenotypic and activity-based profiling of electrophilic fragments
Source: Cell Chem Biol. Author manuscript; Available in PMC 2025 Apr 14. (PMC11995724; doi:10.1016/j.chembiol.2025.02.001)
Supplement: MMC1 [file NIHMS2061764-supplement-MMC1.pdf]

**Cell Chemical Biology, Volume *32***

## Supplemental information

# Antibiotic target discovery by integrated phenotypic and activity-based profiling of electrophilic fragments

**Yizhen Jin, Sadhan Jana, Mikail E. Abbasov, and Hening Lin**

## Supplementary Tables

**Table S1.** Hit count with different warhead scaffolds. Related to Figure 1.

| Warhead | Total amount in Cys-library | Hit amount (SA) | Hit amount (VC) |
|---|---|---|---|
| 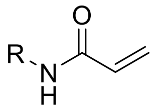 | 960 | 1 | 1 |
| 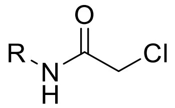 | 751 | 11 | 0 |
| 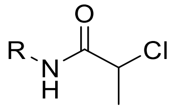 | 529 | 0 | 0 |
| 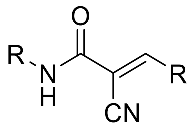 | 640 | 4 | 1 |
| 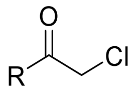 | 210 | 31 | 15 |
| 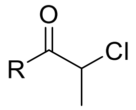 | 26 | 0 | 0 |
| 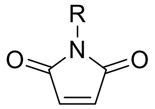 | 56 | 1 | 0 |
| 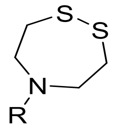 | 25 | 0 | 0 |
| 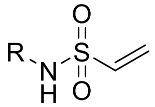 | 1 | 0 | 0 |
| 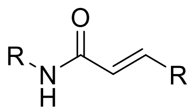 | 2 | 0 | 0 |

SA: *S. aureus*, VC: *V. cholerae*

**Table S2.** MIC table of negative control compounds. Related to Table 1.

| Structure | Compound Name | MIC (µM) | | | |
|---|---|---|---|---|---|
| | | ***S. flexneri*** | ***V. cholerae*** | ***E. coli*** | ***S. aureus*** |
| 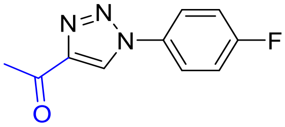 | 10-F05-N | >100 | >100 | >100 | >100 |
| 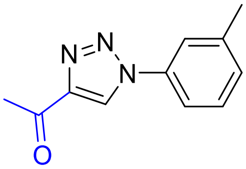 | 10-L07-N | >100 | >100 | >100 | >100 |
| 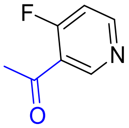 | 10-I09-N | >100 | >100 | >100 | >100 |
| 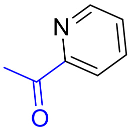 | 10-J03-N | >100 | >100 | >100 | >100 |

**Table S3.** Primer list. Related to STAR methods.

| Primer Name | Sequence |
|---|---|
| Genomic primer for sequencing | |
| SF_FabH_FWD | ATTTCATACTGAAATTTGACGATAATAAAG |
| SF_FabH_REV | CAGCGAACTCGCAGTTTGCAAGTGA |
| SF_MiaA_FWD | CACGTTCCCGACGCAGTGCGTTCAG |
| SF_MiaA_REV | AGCGAACATGCGCAGTGGTCAATGG |
| SF_PdxY_FWD | CGTCTGTTTGGTCGTTTTACCTTAC |
| SF_PdxY_REV | GTTAGAGCACATTGCGGCATTTATG |
| SA_FabH_FWD (also used for qPCR) | ATTTCATACTGAAATTTGACGATAATAAAG |
| SA_FabH_REV | ATCGATACGTGTAATTTTATCGATACCATT |
| SF_YiiD_FWD | GAACAGTGATGTAGCAAGACACGTATGGTG |
| SF_YiiD_REV | CAGCAAGAGATGAACACGCAAACAGGA |
| SA_SAS0989_FWD (also used for qPCR) | AATAGAATTGATGTCAATTTAAGAATTGGTTTATTACAAA |
| SA_SAS0989_REV | GTTATTCTATGTTATTATTAGTAAATTAAATGTAAATGATT |
| SA_SAS1368_FWD (also used for qPCR) | CAGAAACATCCTCCTGCTAATGTTGCATAT |
| SA_SAS1368_REV | CTTTCCACCTTATACATTTGAAGACTGGGAA |
| qPCR | |
| SA_FabH_qPCR_REV | AGATGTATCTAAAAATTGCTCAAAATAGGC |

| | |
|---|---|
| SA_SAS0989_YiiD analog_qPCR_REV | TCATTAGCTAAAGATGAAGGTTTTTACGT |
| SA_SAS13698_YiiD analog 2_qPCR_REV | TGAAGCTTTTCATGTGAATAATTACGATAT |
| SA_araC1_qPCR_FWD | AGAAATGGAAGTTACAGATGAAGTATTCG |
| SA_araC1_qPCR_REV | TCGCAGTATTTTTAACAAGCTCGAG |
| SA_aroE_qPCR_FWD | AACGTATCATCTATTTGTTTAATCATATTT |
| SA_aroE_qPCR_REV | TTGTTTACCAAGGTGCGGAAAGCT |
| Mutagenesis | |
| SA_FabH_C112S_FWD | GGATCAACTTGCAGCATCTTCTGGATTTA |
| SA_FabH_C112S_REV | TAAATCCAGAAGATGCTGCAAGTTGATCC |

Other supplementary tables are contained in an Excel file with several tabs. The contents are listed below.

**Table S4.** Screening Results for the Cys-library. Related to Figure 1 and STAR Methods.

**Table S5.** MIC tables. Related to Figure 2 and Table S3.

**Table S6.** Processed ABPP Result. Related to Figure 3.

**Table S7.** P19 MSSA476 WGS Variants table. Related to Figure 4.

## Supplementary Figures

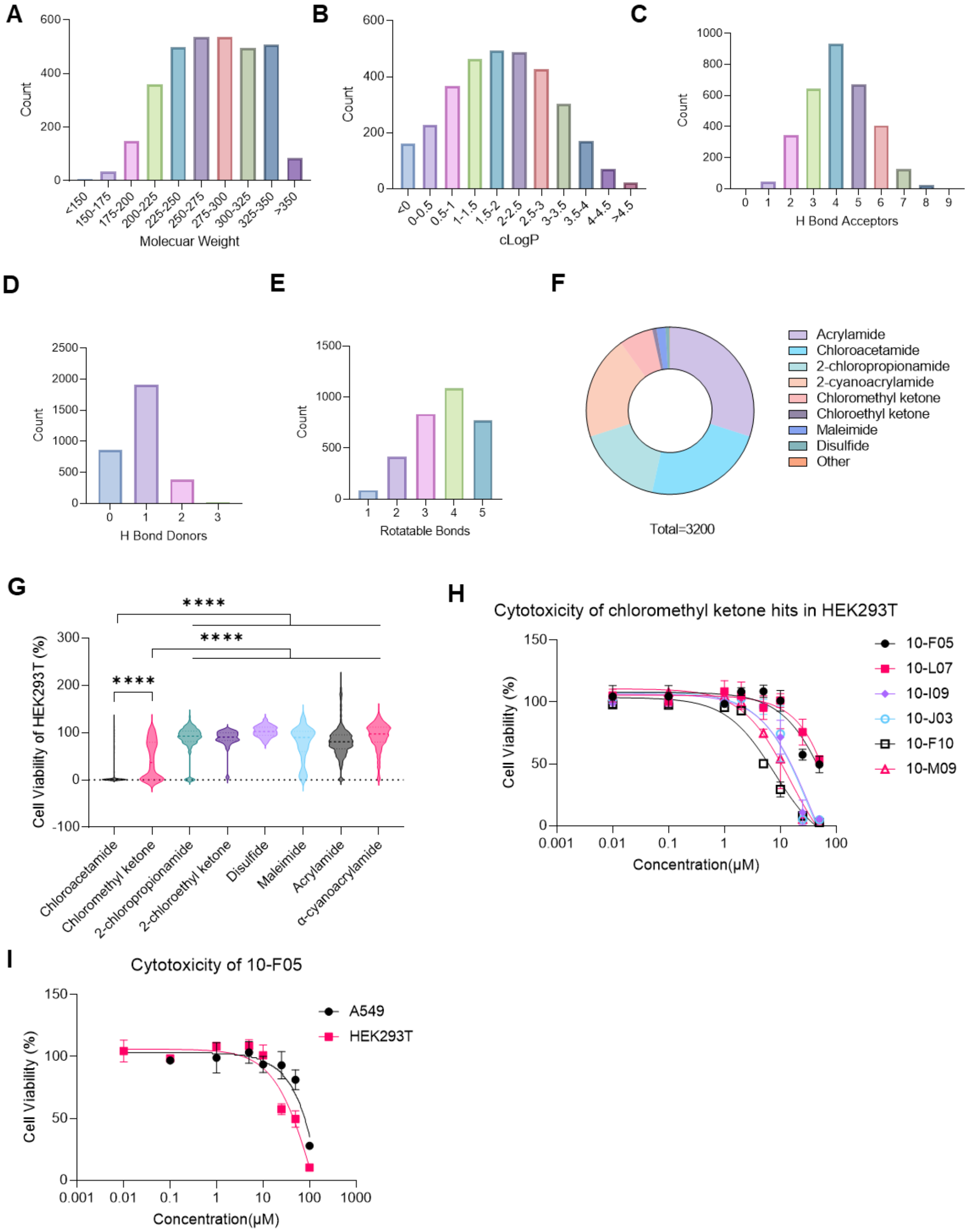


**Figure S1.** Molecular properties of the Cys-library. Related to Figure 1. (**A**) Molecular weight. (**B**) cLogP. (**C**) H bond acceptors. (**D**) H bond donors. (**E**) Rotatable bonds. (**F**) Warhead distribution of the Cys-library. All the properties were calculated using Data Warrior. (**G**) Cytotoxicity profiling of the Cys-library at 25 µM. Cell viability of HEK293T cells was measured using the CellTiter-Glo 2.0 cell viability assay kit after a two-day incubation period with the Cys-library. Each group was

compared to chloroacetamide and chloromethyl ketone scaffolds using unpaired two-tailed t-tests. **** P <0.0001. (**H**) Cytotoxicity profiling of 10-F05 derivatives in HEK293T cells. (**I**) Cytotoxicity of 10-F05 in A549 and HEK293T cells. Cell viability was assessed using the CellTiter-Glo 2.0 cell viability assay kit after a two-day incubation period. Data was plotted using mean ± SD.

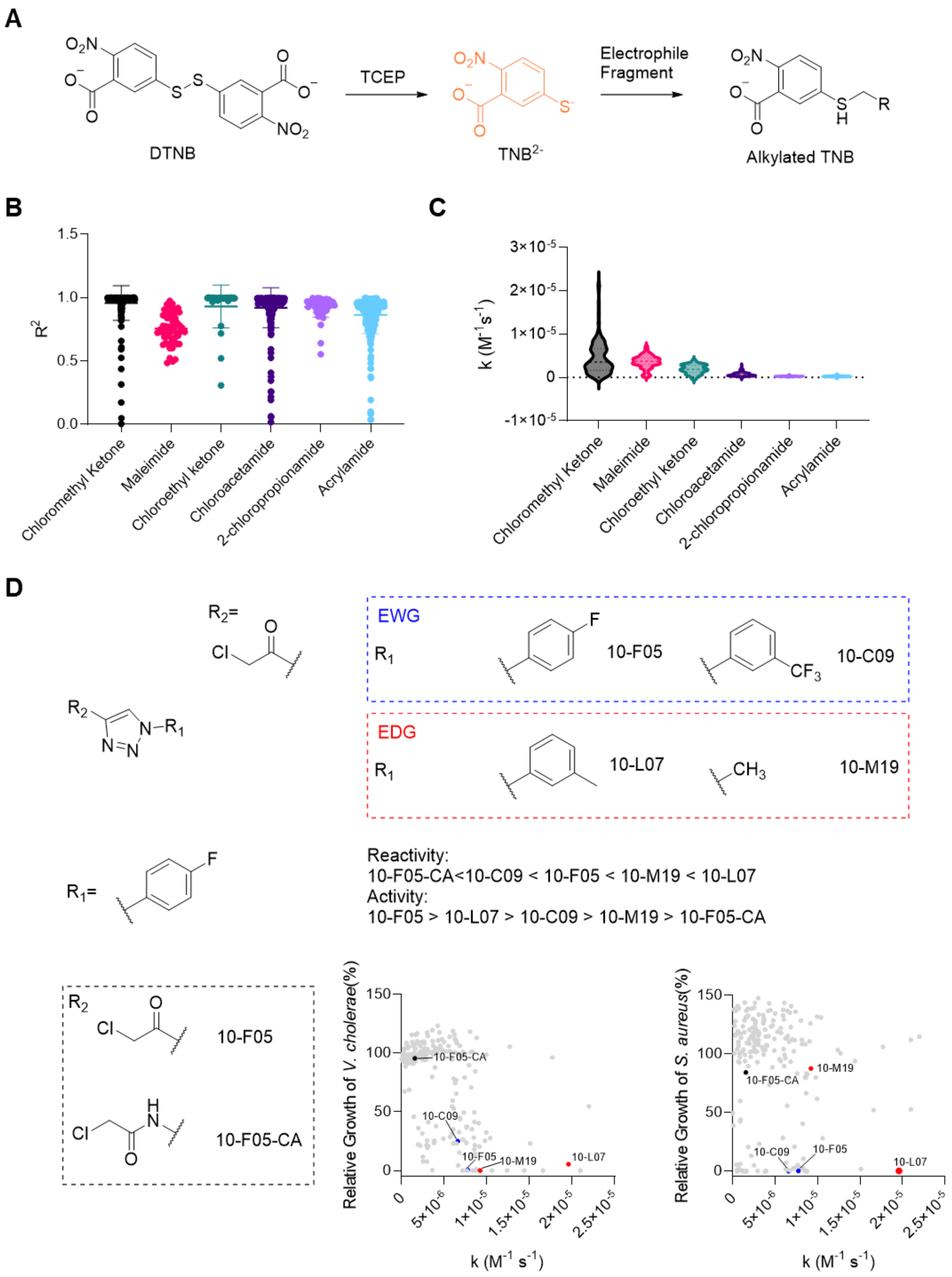


**Figure S2.** Reactivity profiling of Cys-library. Related to STAR Methods. (**A**) Scheme showing the reactions of the reduced DTNB reactivity assay. (**B**) $R^2$ of tested warheads. $R^2$ indicates how well the reaction data fits the second-order reaction rate equation for different warheads (mean ± SD). (**C**) Reactivity of tested warheads in Cys-library. (**D**) Antibacterial activity and reactivity of

triazole ring conjugated chloromethyl ketones hits.  More reactive compounds do not necessarily have higher antibacterial activities.

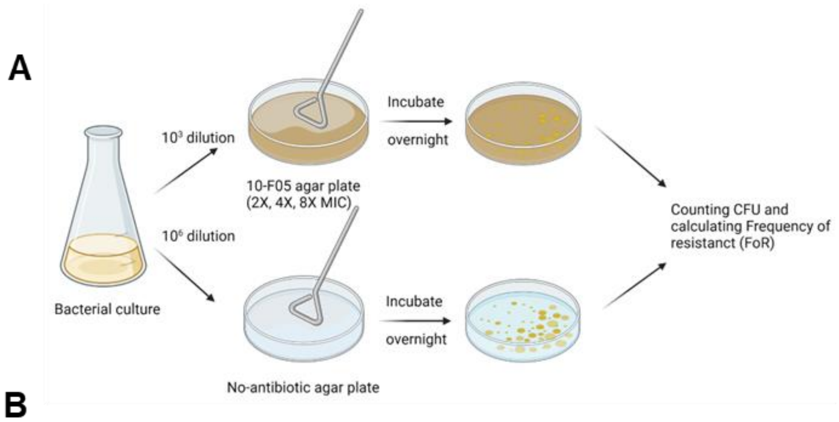


B

| Strain | Strating CFU(mL) | Frequency of resistance (%) | | |
|---|---|---|---|---|
| | | 2X MIC | 4X MIC | 8X MIC |
| *S. flexneri* M90T | ~4 × $10^9$ | < limit of detection | < limit of detection | < limit of detection |
| *S. aureus* MSSA476 | ~9 × $10^9$ | ~0.05 | ~0.05 | < limit of detection |

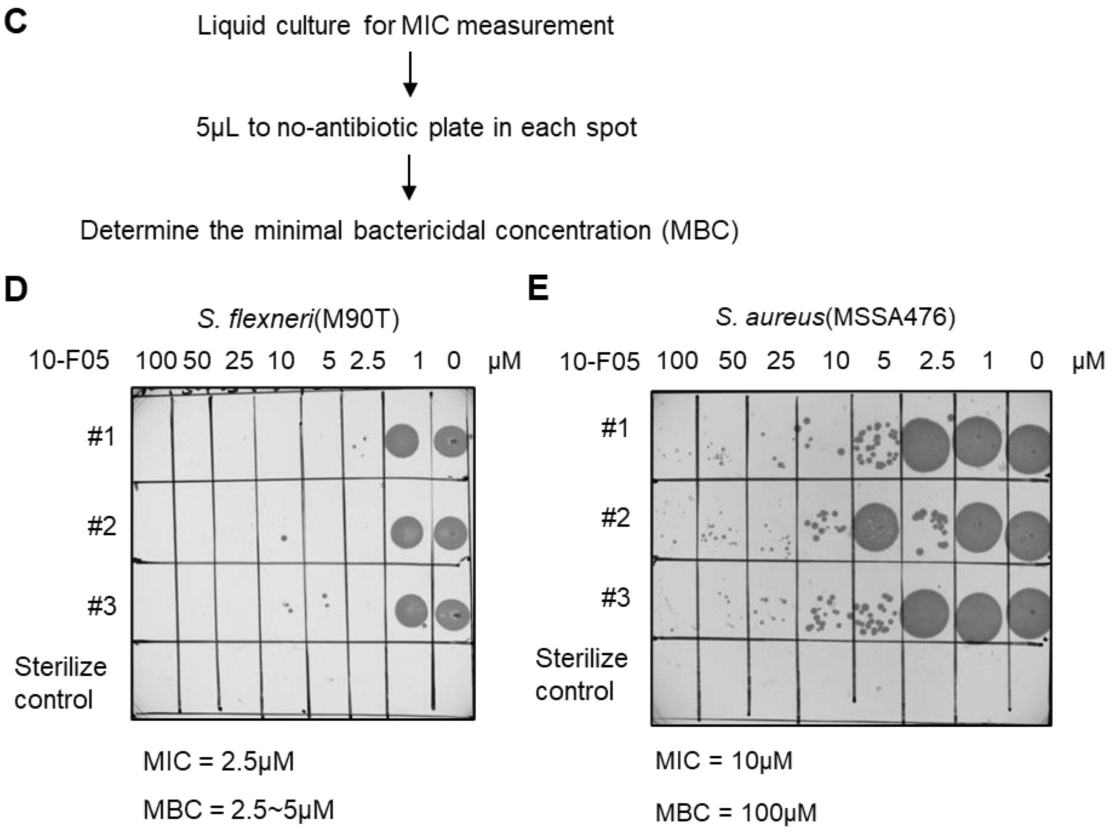


**Figure S3.** Frequency of resistance of *S. flexneri* M90T and *S. aureus* MSSA476 against 10-F05. Related to Figure 2. (**A**) Workflow of determining frequency of resistance. 10-F05 containing agar

plates were prepared using three concentrations (2X, 4X, 8X MIC). (**B**) Frequency of resistance in *S. flexneri* M90T and *S. aureus* MSSA476 in three different concentrations of 10-F05 agar plate. 10-F05 is bactericidal in *S. flexneri* M90T and bacteriostatic in *S. aureus* MSSA476. Related to Figure 2. (**C**) Workflow for measuring MBC. (**D**) (**E**) Spot plate for determining MBC of 10-F05 in *S. flexneri* M90T and *S. aureus* MSSA476, respectively.

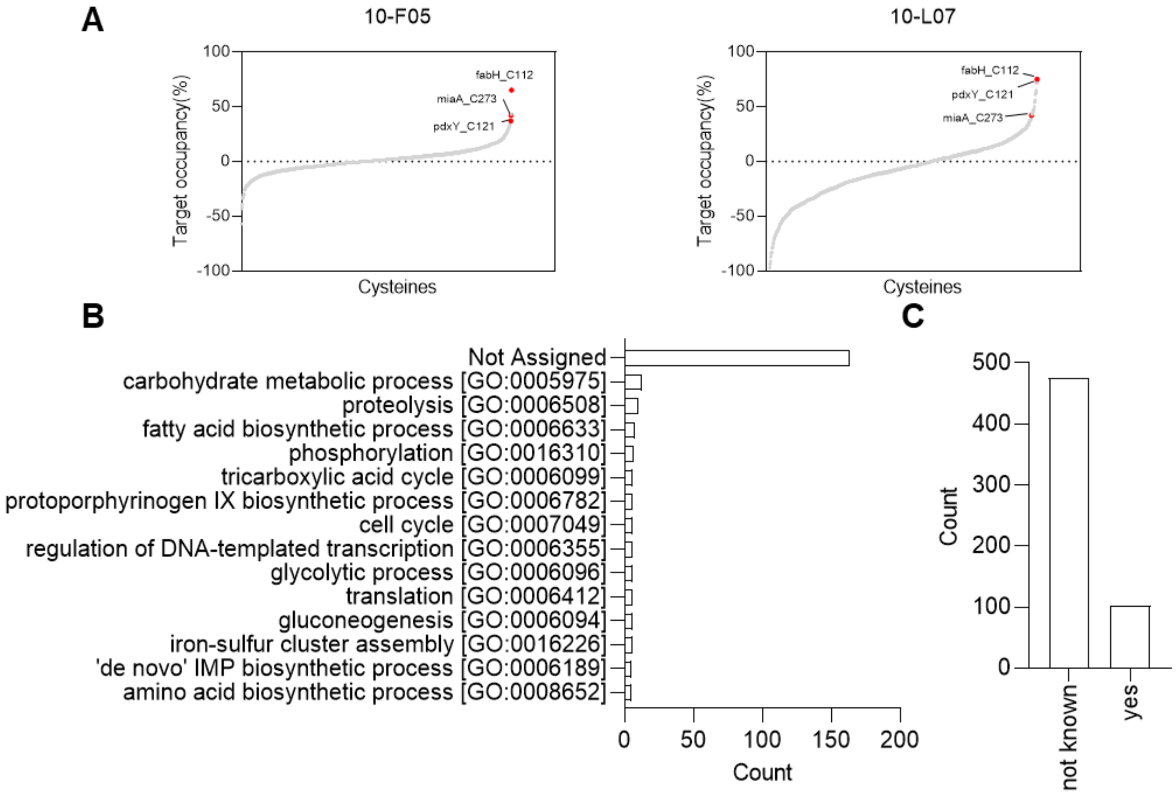


**Figure S4.** ABPP results of 10-F05 and 10-L07. Related to Figure 3. (**A**) Target occupancy profile of 10-F05 and 10-L07 against 1035 identified cysteines. Selected cysteines are highlighted in red. (**B**) Count of quantified protein function for the protein hits identified in the proteomics study. Protein functions were queried from UniProt Database. (**C**) Count of likely essential proteins for the protein hits identified in the proteomics study. Protein essentiality was predicted by NetGene database.

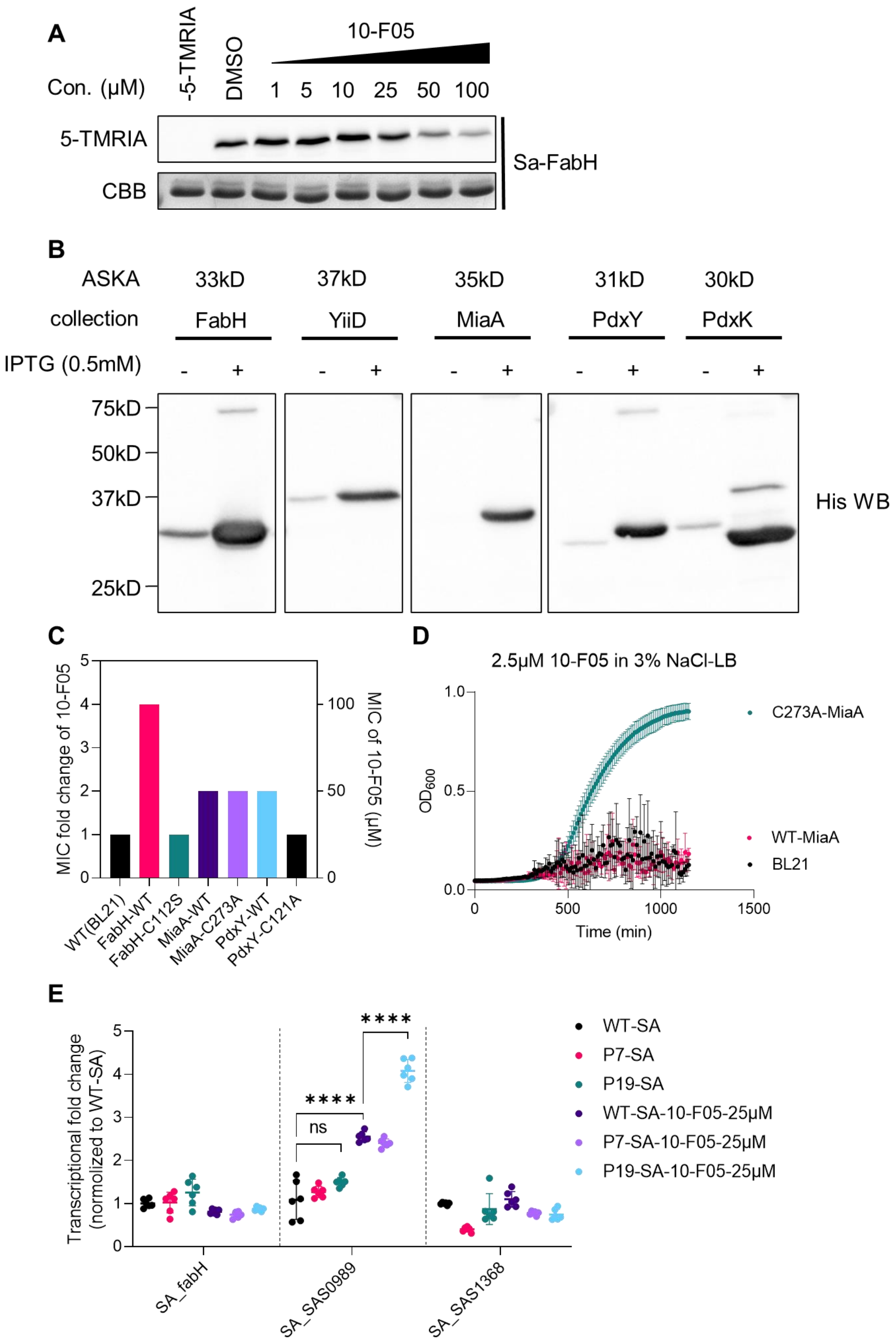

A
10-F05
-5-TMRIA
DMSO
Con. (μM)
1 5 10 25 50 100
5-TMRIA
CBB
Sa-FabH
B
ASKA collection
33kD FabH
37kD YiiD
35kD MiaA
31kD PdxY
30kD PdxK
IPTG (0.5mM)
- + - + - + - + - +
75kD
50kD
37kD
25kD
His WB
C
MIC fold change of 10-F05
MIC of 10-F05 (μM)
WT(BL21)
FabH-WT
FabH-C112S
MiaA-WT
MiaA-C273A
PdxY-WT
PdxY-C121A
D
2.5μM 10-F05 in 3% NaCl-LB
OD600
Time (min)
C273A-MiaA
WT-MiaA
BL21
E
Transcriptional fold change (normolized to WT-SA)
ns
****
****
SA_fabH
SA_SAS0989
SA_SAS1368
WT-SA
P7-SA
P19-SA
WT-SA-10-F05-25μM
P7-SA-10-F05-25μM
P19-SA-10-F05-25μM

**Figure S5.** Validation of 10-F05 targets. Related to Figure 4. (**A**) In-gel fluorescence labeling of *S. aureus* FabH protein using 5-TMRIA competed by 10-F05 pre-incubation at indicated concentrations. (**B**) Verification of ASKA overexpression strains. IPTG (0.5 mM) was added into the cell culture to induce protein overexpression. Protein molecular weight, FabH: 33kD; YiiD: 37kD; MiaA: 35kD; PdxY: 31kD; PdxK: 30kD. (**C**) MIC fold change of 10-F05 tested in target gene overexpression BL21 strains. (**D**) Growth curve of BL21 *E. coli* with or without overexpressing WT or C273A MiaA cultured in 3% NaCl-LB media with 2.5 µM of 10-F05. Data was plotted using mean ± SD. (**E**) Transcriptional level fold changes of *S. aureus fabH*, SAS0989, and SAS1368 from three passages obtained in the resistance development assay. qPCR quantification was performed on WT, P7, and P19 *S. aureus* strains from Figure 2C. The bacteria were treated with either DMSO or 25 µM of 10-F05 for 2 hours before being collected for qPCR analysis. qPCR primers were listed in the supplementary data. The p values of unpaired two-tailed t-tests were performed in GraphPad Prism (version 9.3.1). Data was plotted using mean ± SD, ****p $< 0.0001$, and ns, not significant.

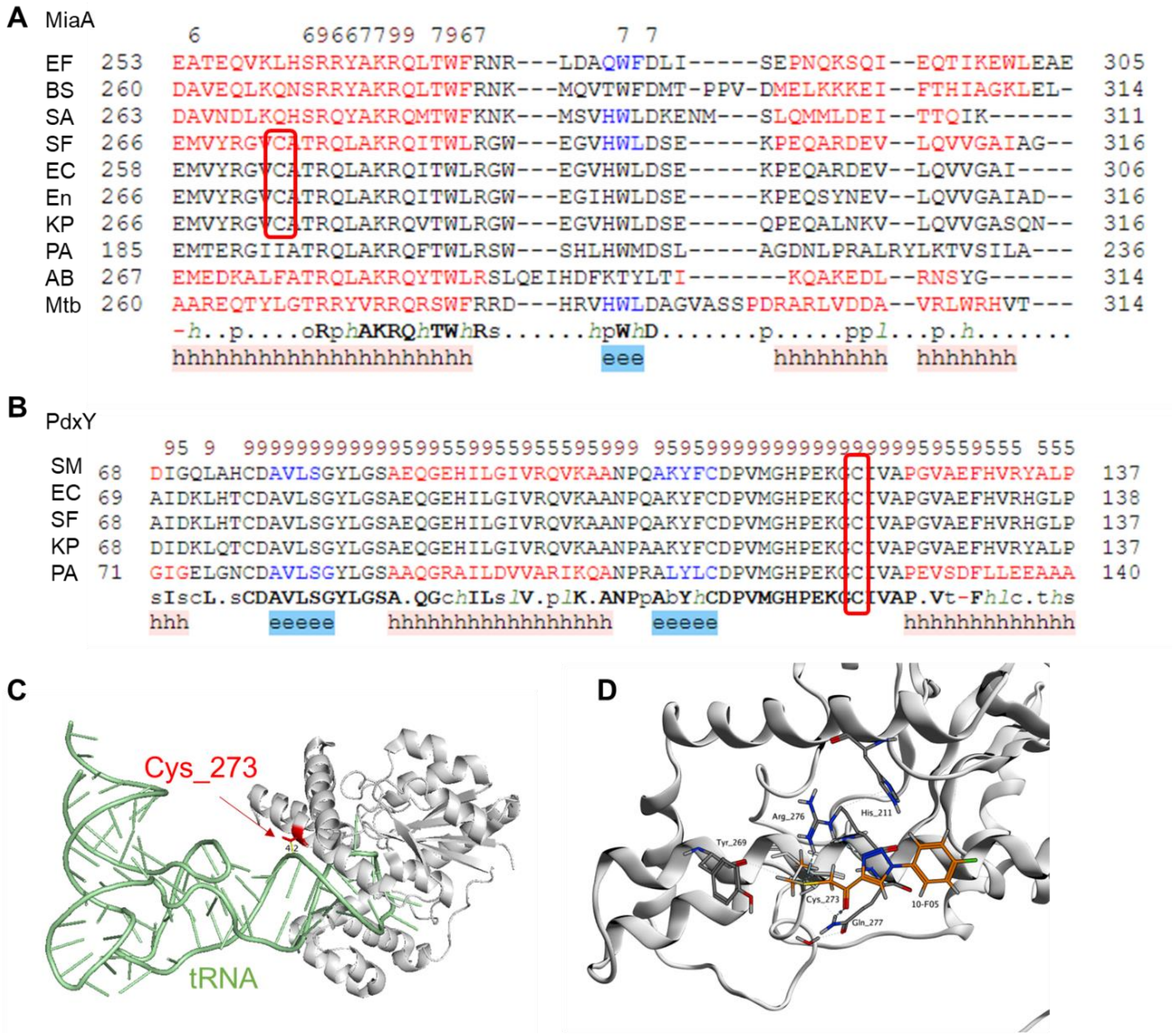

**Figure S6.** Structure-based sequence alignment. Related to Figure 5. (**A**) MiaA. (**B**) PdxY. EF: *E. faecium* (AF-A0A7V7GPP3-F1-model_v4); BS: *B. subtilis* strain 168 (AF-O31795-F1-model); SA: *S. aureus* MSSA476 (AF-Q6G9R0-F1-model_v4); SF: *S. flexneri* M90T (AF-A0A4P7TQH3-F1-model_v4); EC: *E. coli* K12 (PDB: 2ZM5); En: *Enterobacter* sp. strain 638 (AF-A4W5R2-F1-model_v4); KP: *K. pneumoniae* strain 342 (AF-B5Y333-F1-model_v4). PA: *P. aeruginosa* ATCC15692 (PDB: 3CRM); AB: *A. baumannii* BAA1790 (AF-A0A059ZUH9-F1-model_v4); Mtb: *M. tuberculosis* ATCC 25177 (AF-A5U679-F1-model_v4). PdxY, SM: *S. typhimurium* strain LT2 (AF-Q8ZPM8-F1-model_v4); EC: *E. coli* K12 (PDB: 1TD2); SF: *S. flexneri* M90T (AF-A0A4P7TT64-F1-model_v4); KP: *K. pneumoniae* IS53 (AF-W1EDE7-F1-model_v4); PA: *P. aeruginosa* PAO1 (PDB: 5B6A). The covalently targeted cysteines are highlighted in red boxes. Structural insights of MiaA and tRNA substrate. (**C**) MiaA Cys273 is very close to the bound tRNA substrate. *E. coli* MiaA (PDB: 2ZM5). (**D**) Predicted covalent interaction of 10-F05 with nearby amino acids residues. Hydrogen bonding interactions are indicated by short blue sticks.

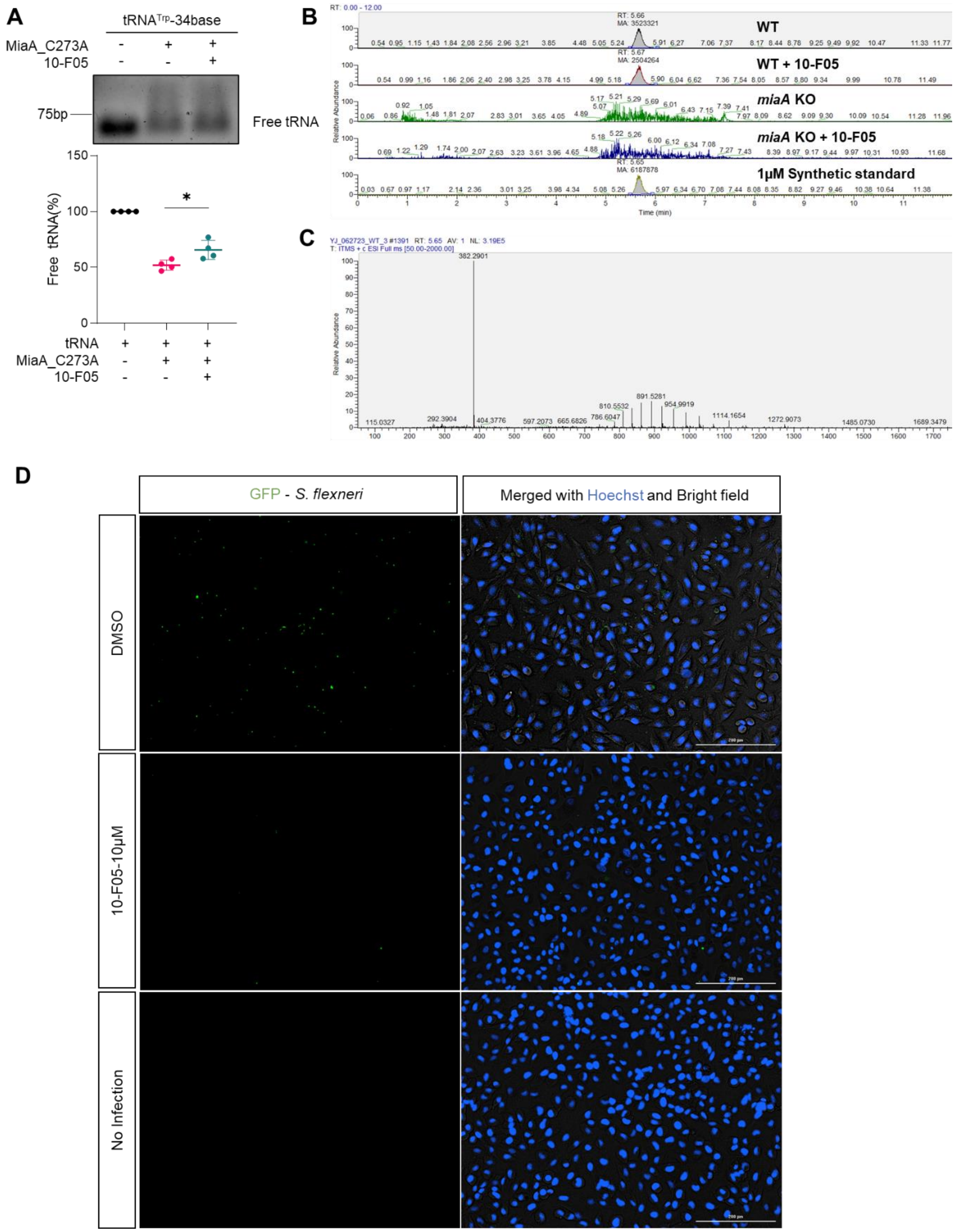


**Figure S7.** Validation of 10-F05's inhibitory activity on MiaA. Related to Figure 5 and Figure 6. (**A**) 10-F05 only slightly decreases MiaA_C273A binding with $tRNA^{Trp}$-34. $^{*}p < 0.05$. (**B**) Representative images of $ms^2i^6A$ peak in LC-MS analysis. Peak corresponding to the $ms^2i^6A$ was searched using ms/z range from 381.5000~382.5000. (**C**) MS spectrum of the $ms^2i^6A$ peak. (**D**)

Representative images for GFP-labeled *S. flexneri* infection assays in Hela cells. GFP tagged *S. flexneri* M90T was treated with 10 µM of 10-F05 for 1 h before infection. Live cells were stained by Hoechst dye. Scale bar: 200 µm.
