# Supplementary material for: Antibiotic target discovery by integrated phenotypic and activity-based profiling of electrophilic fragments
Source: Cell Chem Biol. Author manuscript; Available in PMC 2025 Apr 14. (PMC11995724; doi:10.1016/j.chembiol.2025.02.001)

Data S1. ^{1}H-NMR data for 10-F05-CA, related to STAR methods.

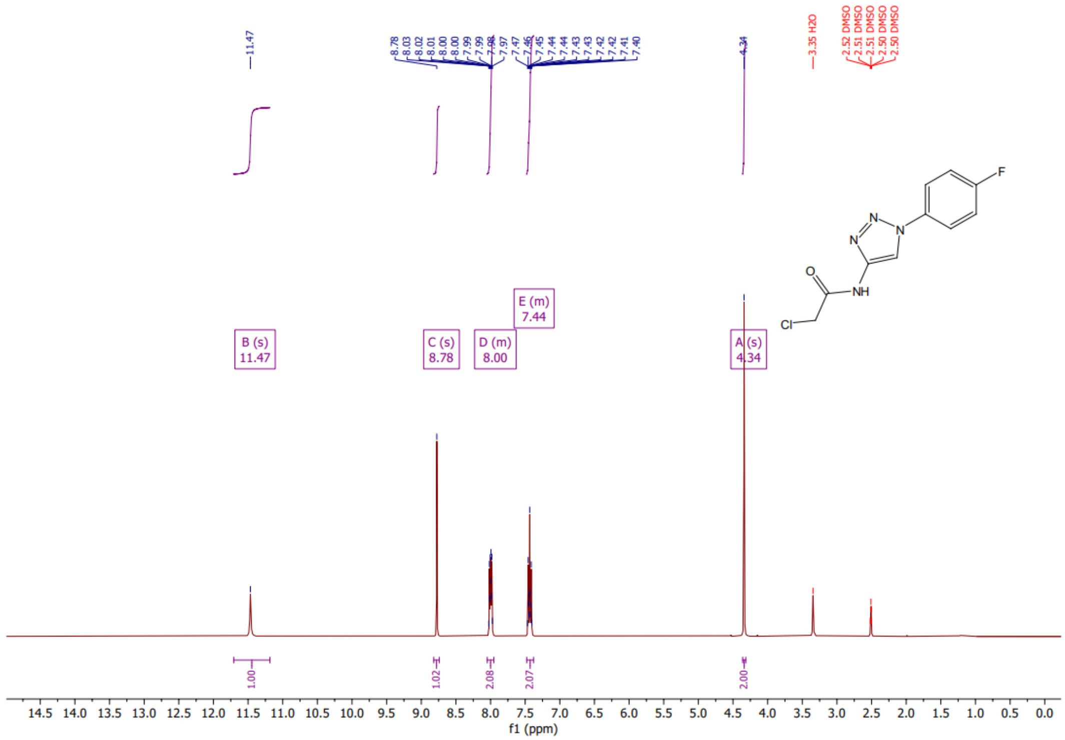

^{13}C-NMR data for 10-F05-CA, related to STAR methods.

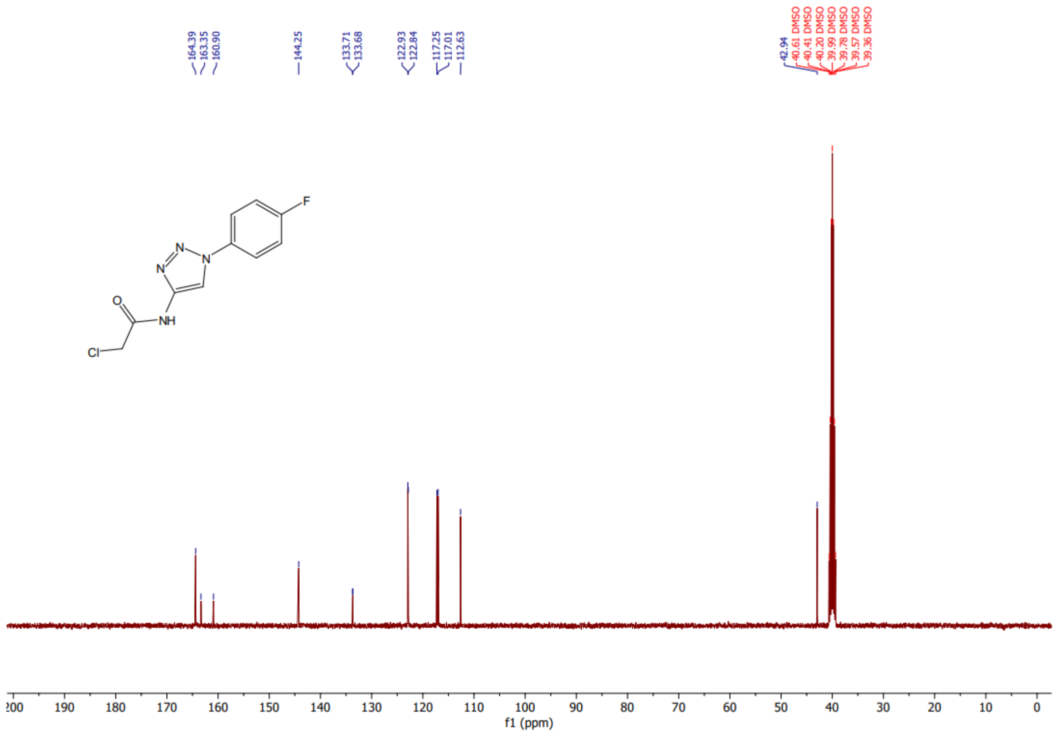

Supplement: MMC6 [file NIHMS2061764-supplement-MMC6.pdf]
